# Cyclonerane Derivatives from the Algicolous Endophytic Fungus *Trichoderma asperellum* A-YMD-9-2

**DOI:** 10.3390/md17050252

**Published:** 2019-04-28

**Authors:** Yin-Ping Song, Feng-Ping Miao, Xiang-Hong Liu, Xiu-Li Yin, Nai-Yun Ji

**Affiliations:** 1 Yantai Institute of Coastal Zone Research, Chinese Academy of Sciences, Yantai 264003, China; ypsong@yic.ac.cn (Y.-P.S.); fpmiao@yic.ac.cn (F.-P.M.); xianghongliu@yic.ac.cn (X.-H.L.); xlyin@yic.ac.cn (X.-L.Y.); 2 College of Resources and Environment, University of Chinese Academy of Sciences, Beijing 100049, China; 3 Center for Ocean Mega-Science, Chinese Academy of Sciences, Qingdao 266071, China

**Keywords:** *Trichoderma asperellum*, sesquiterpene, cyclonerane

## Abstract

Seven previously unreported cyclonerane derivatives, namely, 3,7,11-trihydroxycycloneran-10-one, cycloneran-3,7,10,11-tetraol, cycloneran-3,7,11-triol, 11,12,15-trinorcycloneran-3,7,10-triol, 7,10*S*-epoxycycloneran-3,15-diol, 7,10*R*-epoxycycloneran-3,15-diol, and (10*Z*)-15-acetoxy-10-cycloneren-3,7-diol, were isolated in addition to the known (10*Z*)-cyclonerotriol, (10*E*)-cyclonerotriol, catenioblin C, and chokol E from the culture of *Trichoderma asperellum* A-YMD-9-2, an endophytic fungus obtained from the marine red alga *Gracilaria verrucosa*. The structures of previously unreported compounds were established by spectroscopic techniques, including 1D/2D NMR, MS, and IR. The isolation of these new cyclonerane derivatives greatly adds to the structural diversity of unusual cyclonerane sesquiterpenes, and several isolates exhibit potent inhibition against some marine phytoplankton species.

## 1. Introduction

**Figure 1:**
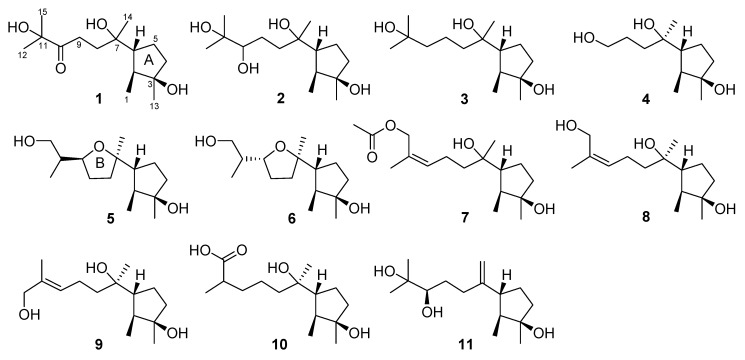
Structures of **1**–**11** (the stereochemistry in **1**–**8** and **10** only represents relative configuration).

*Trichoderma* species have proven to be prolific sources of diterpenes and sesquiterpenes, especially some unusual terpene skeletons, such as harziane, proharziane, wickerane, citrinovirin, trichaspin, and cyclonerane [1,2,3,4,5,6,7,8]. Of the cyclonerane-type sesquiterpenes, cyclonerodiol and its analogs have been isolated from several species of *Trichoderma* [4,5,6,9,10,11,12,13,14]. To date, around 30 cyclonerane sesquiterpenes have been characterized, and almost half of them occurred in *Trichoderma* species. In our ongoing search for new and bioactive metabolites from marine algicolous fungi [15], an endophytic strain (A-YMD-9-2) of *Trichoderma asperellum*, isolated from the marine red alga *Gracilaria verrucosa*, was chemically examined. As a result, seven new cyclonerane derivatives, namely, 3,7,11-trihydroxycycloneran-10-one (**1**), cycloneran-3,7,10,11-tetraol (**2**), cycloneran-3,7,11-triol (**3**), 11,12,15-trinorcycloneran-3,7,10-triol (**4**), 7,10*S*-epoxycycloneran-3,15-diol (**5**), 7,10*R*-epoxycycloneran-3,15-diol (**6**), and (10*Z*)-15-acetoxy-10-cycloneren-3,7-diol (**7**), together with four known compounds, namely, (10*Z*)-cyclonerotriol (**8**) [16], (10*E*)-cyclonerotriol (**9**) [16,17], catenioblin C (**10**) [18], and chokol E (**11**) [19], were isolated and identified (Figure 1). Herein, the isolation, structure elucidation, and bioactivity of these compounds are described in detail.

## 2. Results and Discussion

**Figure 2:**
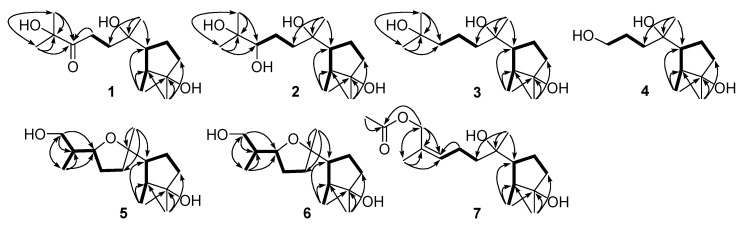
Key COSY (bold lines) and HMBC (arrows) correlations of **1**–**7**.

Compound **1** was obtained as a colorless oil. The molecular formula C_15_H_28_O_4_ was determined by HREIMS *m*/*z* 272.1991 [M]^+^ (calculated for C_15_H_28_O_4_, 272.1988), requiring two degrees of unsaturation. Its IR absorption bands at 3440 and 1713 cm^−1^ suggested the presence of hydroxy and carbonyl groups. In combination with HSQC data, the ^1^H NMR spectrum (Table 1) showed one methyl doublet, four methyl singlets, and an array of signals at δ_H_ 1.5–2.6 for four methylenes and two methines. Aided by the 90° and 135° DEPT experiments, 15 resonances in the ^13^C NMR spectrum (Table 2) were assigned to five methyls, four methylene sp^3^, two methine sp^3^, three quaternary sp^3^ and one quaternary sp^2^ carbons. The ^13^C NMR spectrum of **1** exhibited the signals at δ_C_ 79.4 and 215.1, attributed to an oxygenated quaternary sp^3^ carbon and a ketone carbonyl group, respectively, instead of the signals for the vinyl group in cyclonerodiol [20]. HMBC correlations from H_3_-12 (δ_H_ 1.30) to C-10 (δ_C_ 215.1), C-11 (δ_C_ 79.4), and C-15 (δ_C_ 28.0) and from H_3_-15 (δ_H_ 1.29) to C-10, C-11, and C-12 (δ_C_ 27.8) established the connectivity between C-10 and C-11, which then extended to C-8 (δ_C_ 31.5) as corroborated by the HMBC correlation from H_2_-9 (δ_H_ 2.51) to C-10 as well as by the COSY correlation from H_2_-8 (δ_H_ 2.12 and 1.89) to H_2_-9. The connectivity around ring A was deduced to be the same as those of cyclonerodiol based on their identical NMR data, and this was confirmed by the COSY correlations from H_3_-1 (δ_H_ 1.03)/H-2 (δ_H_ 1.56)/H-6 (δ_H_ 1.97)/H_2_-5 (δ_H_ 1.88 and 1.63)/H_2_-4 (δ_H_ 1.67 and 1.56) and HMBC correlations from H_3_-1 to C-2 (δ_C_ 44.9), C-3 (δ_C_ 81.4), and C-6 (δ_C_ 55.1), from H_3_-13 (δ_H_ 1.25) to C-2, C-3, and C-4 (δ_C_ 40.4), and from H_3_-14 (δ_H_ 1.19) to C-6, C-7 (δ_C_ 76.0), and C-8 (δ_C_ 31.5) (Figure 2). The NOESY correlation from H_3_-1 to H-6 further verified that Me-1 was *syn* to H-6, while the NOESY correlations from H_3_-1 to H-5a (δ_H_ 1.88) and from H_3_-13 to H-5b (δ_H_ 1.63) suggested Me-1 to be opposite to Me-13. The above information evidenced **1** to be 3,7,11-trihydroxycycloneran-10-one.

Compound **2** was isolated as a colorless oil, and its molecular formula was established as C_15_H_30_O_4_, which has two more hydrogen atoms than that of **1**, based on the (+)-HREIMS *m*/*z* 297.2037 [M + Na]^+^ (calculated for C_15_H_30_O_4_Na, 297.2042). A detailed comparison of its NMR signals (Table 1 and Table 2) with those of **1** revealed the presence of ring A and its affiliated groups in **2**. In addition, this compound exhibited a deshielded broad doublet (*J* = 10.3 Hz) at δ_H_ 3.38 in the ^1^H NMR spectrum. Combined with the HSQC correlation of this proton signal to the carbon signal at δ_C_ 79.0, this signal was attributed to the oxymethine proton on C-10. This was supported by the HMBC correlations from oxymethine sp^3^ carbon at δ_C_ 79.0 (C-10) to H_3_-12 (δ_H_ 1.16) and H_3_-15 (δ_H_ 1.21) as well as a COSY correlation from H-10 to H_2_-9 (δ_H_ 1.61 and 1.40) (Figure 2). Thus, **2** was deduced to be cycloneran-3,7,10,11-tetraol. Previously, cyclonerodiol B from the mangrove-derived endophytic fungus *Trichoderma* sp. Xy24 was assigned the same structure as **2**, but it should be revised to epicyclonerodiol oxide in view of their identical NMR data [9,13]. To confirm the absolute configuration at C-10, Mosher’s reactions were performed under the conditions described previously [21]. Unfortunately, no esterified products were obtained after the purification via preparative thin-layer chromatography (TLC).

Compound **3** was purified as a colorless oil, and its HREIMS *m*/*z* 258.2198 [M]^+^ (calculated for C_15_H_30_O_3_, 258.2195) produced the molecular formula C_15_H_30_O_3_, which has one oxygen atom less than that of **2**. Its ^13^C NMR spectrum (Table 2) showed a close similarity to that of **2**, except for the lack of the signal for an oxymethine group and the presence of the signal for a methylene group of C-10 (δ_C_ 44.5), which was supported by its HMBC correlations to H_3_-12 (δ_H_ 1.22) and/or H_3_-15 (δ_H_ 1.22). Other HMBC and COSY correlations, as shown in Figure 2, further confirmed **3** to be cycloneran-3,7,11-triol, a 10-deoxy derivative of **2**.

Compound **4** was also isolated as a colorless oil. A molecular formula C_12_H_24_O_3_ was assigned by HREIMS *m*/*z* 216.1724 [M]^+^ (calculated for C_12_H_24_O_3_, 216.1725), implying one degree of unsaturation. Its ^1^H and ^13^C NMR data (Table 1 and Table 2) resembled those of **2**, except for the lack of signals for a 2-hydroxy-2-propyl group (δ_H_ 1.16 and 1.21, δ_C_ 23.5, 26.7, and 73.4) and the presence of signals for a hydroxymethylene group (δ_H_ 3.68 and δ_C_ 63.6). This group was deduced to be situated at the side chain terminus on the basis of HMBC correlations from H_3_-14 (δ_H_ 1.17) to C-6 (δ_C_ 54.9), C-7 (δ_C_ 74.8), and C-8 (δ_C_ 36.8) and COSY correlations from H_2_-10 (δ_H_ 3.68) to H_2_-9 (δ_H_ 1.68) and from H_2_-9 to H_2_-8 (δ_H_ 1.57). HMBC correlations (Figure 2) from H_3_-1 (δ_H_ 1.05) to C-2 (δ_C_ 44.5), C-3 (δ_C_ 81.4), and C-6, and from H_3_-13 (δ_H_ 1.26) to C-2, C-3, and C-4 (δ_C_ 40.5), and COSY correlations from H_3_-1 to H_2_-4 (δ_H_ 1.69 and 1.56) via H-2 (δ_H_ 1.60), H-6 (δ_H_ 1.87), and H_2_-5 (δ_H_ 1.88 and 1.55), further verified the connectivity of ring A. Thus, **4** was proposed to be 11,12,15-trinorcycloneran-3,7,10-triol, possibly a degradation product of **1** or **2**. To ascertain the relative configuration at C-7, the energy-minimized conformers, regardless of the rotation of the methyl and hydroxy groups as well as the side chain, of 7α- and 7β-methyl isomers optimized at the B3LYP/6-31G(d) level in chloroform, were subjected to the ^13^C NMR calculations using the gauge-independent atomic orbital (GIAO) method at the B3LYP/6-31+G(d,p) level via Gaussian 09 software [22]. Both experimental and calculated shifts were input into Sarotti’s DP4+ sheet (see https://sarotti-nmr.weebly.com) [23], and the calculated data for 7α- and 7β-methyl isomers displayed 73.74% and 26.26% DP4+ probabilities, respectively. Thus, the methyl group at C-7 was tentatively assigned as α orientation.

**Figure 3:**
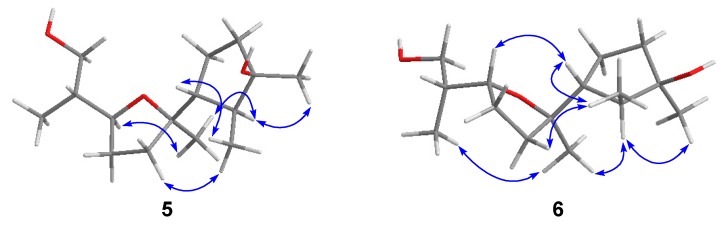
Key NOESY correlations of **5** and **6**.

Compound **5** was obtained as a colorless oil, and the HREIMS spectrum afforded the molecular formula C_15_H_28_O_3_, suggesting two degrees of unsaturation. However, examination of the ^1^H and ^13^C NMR data (Table 1 and Table 2) indicated the absence of any unsaturated bond. Thus, this compound was suggested to possess a bicyclic skeleton. As the NMR data of **5** resembled those of 7,10-epoxycycloneran-3,11,12-triol, the presence of a 7,10-epoxycyclonerane framework was proposed [5]. However, the signal for a methine group appeared in the ^13^C NMR spectrum of **5** instead of that for an oxygenated quaternary carbon in the ^13^C NMR spectrum of 7,10-epoxycycloneran-3,11,12-triol. The fact that the methine group was located at C-11 (δ_C_ 38.2) of **5** was corroborated by its HMBC correlations with H_3_-12 (δ_H_ 0.93) and H_2_-15 (δ_H_ 3.65 and 3.61). HMBC correlations (Figure 2) from H_3_-12 and H_2_-15 to C-10 (δ_C_ 80.3), from H_3_-1 (δ_H_ 1.02) to C-2 (δ_C_ 45.4), C-3 (δ_C_ 81.3), and C-6 (δ_C_ 54.2), from H_3_-13 (δ_H_ 1.24) to C-2, C-3, and C-4 (δ_C_ 40.5), and from H_3_-14 (δ_H_ 1.14) to C-6, C-7 (δ_C_ 85.7), and C-8 (δ_C_ 35.7) further confirmed the structure of **5** as 11-deoxy derivative of 7,10-epoxycycloneran-3,11,12-triol. Additionally, H-10 (δ_H_ 4.07) and Me-14 (δ_H_ 1.14) were on the same face of ring B by their NOESY correlation (Figure 3). The NOESY correlations from H_3_-1 to H-5a (δ_H_ 1.89) and H-6 (δ_H_ 1.95) and from H_3_-13 to H-5b (δ_H_ 1.49) validated the relative configurations around ring A. However, the relative configuration at C-11 remains unsolved.

Compound **6** was isolated as a colorless oil and has the same molecular formula as that of **5** by HREIMS *m*/*z* 256.2028 [M]^+^ (calcd for C_15_H_28_O_3_, 256.2038). Its ^1^H and ^13^C NMR data (Table 1 and Table 2) also resembled those of **5**, except for the deshielded signal for C-10 (δ_C_ 84.2). Thus, **6** was deduced to be a C-10 epimer of **5**, as supported by the NOESY correlation from H_3_-12 (δ_H_ 0.90) to H_3_-14 (δ_H_ 1.17). The similar chemical shifts of C-7 (δ_C_ 86.0) and C-10 (δ_C_ 84.2) to those of epicyclonerodiol oxide further evidenced the opposite orientation of H-10 and C-14 [9]. The relative configurations around ring A and at C-7 were deduced to be the same as those of **5** based on their identical ^1^H and ^13^C NMR data. Moreover, because both C-6 and C-7 are chiral centers, the rotation of the C-6–C-7 single bond is restricted to some extent. Thus, the relative configuration at C-7 could be locked by the NOESY correlations from H_3_-14 to H-2 (δ_H_ 1.54) and H-5b (δ_H_ 1.40) and from H-8a (δ_H_ 1.86) to H_3_-1 (δ_H_ 1.02) and H-2 (Figure 3). As is the case for **5**, the relative configuration at C-11 (δ_C_ 37.4) is not determined.

Compound **7** was obtained as a colorless oil. Its molecular formula was determined to be C_17_H_30_O_4_ by HREIMS (*m*/*z* 298.2138), corresponding to three degrees of unsaturation. The carbonyl signal at δ_C_ 171.3 and the methyl signals at δ_H_ 2.06 and δ_C_ 21.1 as well as their HMBC correlation indicated the presence of an acetyl group. The remaining NMR data (Table 1 and Table 2) showed a close similarity to those of (10*Z*)-cyclonerotriol (**8**) [16]. Thus, **7** was speculated to be an acetylated derivative of **8**, and the acetoxy group was attached to C-15, as evidenced from the HMBC correlation from H_2_-15 (δ_H_ 4.62 and 4.57) to the carbonyl carbon (δ_C_ 171.3). Other HMBC and COSY correlations (Figure 2) further validated the structure of **7**, and the *Z* geometry was corroborated by the NOESY correlations between H_2_-15 and H_2_-9 (δ_H_ 2.16) and between H_3_-12 (δ_H_ 1.74) and H-10 (δ_H_ 5.41).

To develop new inhibitors against harmful microalgae that seriously threaten marine aquaculture, **1**–**11** were evaluated for their growth inhibition against four marine phytoplankton species (*Chattonella marina*, *Heterosigma akashiwo*, *Karlodinium veneficum*, and *Prorocentrum donghaiense*) [24]. The results (Table 3) show that **10**, with IC_50_ values ranging from 1.6 to 2.0 μg/mL, is the most potent to inhibit the four phytoplankton species tested. An analysis of the structure–activity relationship suggested that the carboxyl group at C-15 may greatly contribute to the inhibitory ability of cyclonerane sesquiterpenes. Moreover, the toxicity of **10** to the marine zooplankton *Artemia salina* was also assayed [7], but it does not exhibit any lethal effect at the concentration of 100 μg/mL.

## 3. Materials and Methods

### 3.1. General Experimental Procedures

Optical rotations and ECD curves were determined on a Chirascan CD spectrometer (Applied Photophysics Ltd., Surrey, UK). IR spectra were obtained on a Nicolet iS10 FT-IR spectrometer (Thermo Fisher Scientific, Waltham, MA, USA). NMR spectra were recorded on a Bruker Avance III 500 NMR spectrometer (Bruker Corp., Billerica, MA, USA). Low- and high-resolution EI and ESI^+^ mass spectra were measured on an Autospec Premier P776 mass spectrometer (Waters Corp., Milford, MA, USA) and an Agilent G6230 TOF mass spectrometer (Agilent Technologies Inc., Santa Clara, CA, USA), respectively. Column chromatography (CC) was carried out with silica gel (200–300 mesh, Qingdao Haiyang Chemical Co., Qingdao, China), RP-18 (AAG12S50, YMC Co., Ltd., Kyoto, Japan), and Sephadex LH-20 (GE Healthcare, Uppsala, Sweden). Thin-layer chromatography (TLC) was performed with precoated silica gel plates (GF-254, Qingdao Haiyang Chemical Co., Qingdao, China). Quantum chemical calculations were run with Gaussian 09 software (Gaussian, Inc., Wallingford, CT, USA).

### 3.2. Fungal Material and Fermentation

The fungal strain *Trichoderma asperellum* A-YMD-9-2 (Moniliaceae) was obtained from the inner tissue of the marine red alga *Gracilaria verrucosa* collected from Yangma Island, Yantai, China in August 2016. The fungus was identified by morphological observation and by analysis of the ITS regions of its rDNA, whose sequence data were deposited at GenBank with the accession number MH819724. The fermentation was performed statically at room temperature for 40 days in 200 × 1 L Erlenmeyer flasks, each containing 50 g rice, 0.6 g peptone, 50 mL pure water, and 50 mL natural seawater from the coast of Yantai, China.

### 3.3. Extraction and Isolation

The mycelia and broth were separated by filtration at the end of the fermentation, and the former were dried in the shade and exhaustively extracted with CH_2_Cl_2_ and MeOH (1:1, *v*/*v*). After removing organic solvents under a vacuum, the residue was partitioned between EtOAc and H_2_O to afford an EtOAc-soluble extract (202.1 g). The broth was directly extracted with EtOAc and then concentrated to give an extract (10.3 g). Based on the similar TLC profiles, these two parts were combined and subjected to silica gel CC with step-gradient solvent systems consisting of petroleum ether (PE)/EtOAc (50:1 to 0:1) and then CH_2_Cl_2_/MeOH (10:1 to 0:1) to give eight fractions (Frs. 1–8). Fr. 3 eluted with PE/EtOAc (2:1) and was further purified by CC on RP-18 (MeOH/H_2_O, 3:2) and silica gel (PE/EtOAc, 6:1) to obtain **1** (11.3 mg), **5** (5.2 mg), and **6** (5.9 mg). Fr. 4 eluted with PE/EtOAc (1:1) and was further purified by CC on RP-18 (MeOH/H_2_O, 1:1 to 3:2) and Sephadex LH-20 (MeOH) and preparative TLC (CH_2_Cl_2_/MeOH, 15:1) to yield 7 (8.1 mg), **8** (33.8 mg), **10** (10.1 mg), and **11** (3.6 mg). Fr. 5 eluted with EtOAc and was further purified by CC on RP-18 (MeOH/H_2_O, 2:3 to 3:2) and Sephadex LH-20 (MeOH) and preparative TLC (CH_2_Cl_2_/MeOH, 10:1) to give **2** (26.0 mg), **4** (1.7 mg), and **9** (10.0 mg). Fr. 6 eluted with CH_2_Cl_2_/MeOH (10:1) and was further purified by CC on RP-18 (MeOH/H_2_O, 1:1) and Sephadex LH-20 (MeOH) and preparative TLC (CH_2_Cl_2_/MeOH, 7:1) to produce **3** (12.0 mg).

3,7,11-Trihydroxycycloneran-10-one (**1**): Colorless oil; [α]^20^_D_ − 24 (*c* 0.44, MeOH); IR (KBr) *v*_max_ 3440, 2960, 2932, 1713, 1460, 1663, 1374, 1239, 1125, 1013, 921 cm^−1^; ^1^H and ^13^C NMR data, Table 1 and Table 2 (Figures S1 and S2); 2D NMR data, Figures S3–S6; EIMS *m*/*z* (%) 272 [M]^+^ (<1), 196 (12), 178 (45), 141 (95), 136 (44), 121 (39), 95 (39), 71 (40), 56 (40), 44(100); HREIMS *m*/*z* 272.1991 [M]^+^ (calcd for C_15_H_28_O_4_, 272.1988) (Figure S7).

Cycloneran-3,7,10,11-tetraol (**2**): Colorless oil; [α]^20^_D_ − 30 (*c* 1.0, MeOH); IR (KBr) *v*_max_ 3416, 2967, 1654, 1460, 1378, 1266, 1162, 1074, 1032, 921, 885 cm^−1^; ^1^H and ^13^C NMR data, Table 1 and Table 2 (Figures S8 and S9); 2D NMR data, Figures S10–S13; ESI^+^MS *m*/*z* 297 [M + Na]^+^; HRESI^+^MS *m*/*z* 297.2037 [M + Na]^+^ (calcd for C_15_H_30_O_4_Na, 297.2042) (Figure S14).

Cycloneran-3,7,11-triol (**3**): Colorless oil; [α]^20^_D_ − 18 (*c* 0.62, MeOH); IR (KBr) *v*_max_ 3406, 2965, 1654, 1460, 1377, 1164, 920, 885 cm^−1^; ^1^H and ^13^C NMR data, Table 1 and Table 2 (Figures S15 and S16); 2D NMR data, Figures S17–S20; EIMS *m*/*z* (%) 258 [M]^+^ (<1), 207 (25), 157 (28), 139 (81), 127 (70), 109 (81), 95 (38), 81(45), 44(100); HREIMS *m*/*z* 258.2198 [M]^+^ (calcd for C_15_H_30_O_3_, 258.2195) (Figure S21).

11,12,15-Trinorcycloneran-3,7,10-triol (**4**): Colorless oil; [α]^20^_D_ − 39 (*c* 0.062, MeOH); IR (KBr) *v*_max_ 3426, 2928, 1720, 1635, 1460, 1383, 1054, 920 cm^−1^; ^1^H and ^13^C NMR data, Table 1 and Table 2 (Figures S22 and S23); 2D NMR data, Figures S24–S27; EIMS *m*/*z* (%) 216 [M]^+^ (<1), 165 (11), 157 (11), 139 (79), 103 (31), 96 (40), 85 (98), 81(58), 44(100); HREIMS *m*/*z* 216.1724 [M]^+^ (calcd for C_12_H_24_O_3_, 216.1725) (Figure S28).

7,10S-epoxycycloneran-3,15-diol (**5**): Colorless oil; [α]^20^_D_ +11 (*c* 0.20, MeOH); IR (KBr) *v*_max_ 3426, 2965, 2931, 2876, 1654, 1460, 1376, 1205, 1092, 1024, 919 cm^−1^; ^1^H and ^13^C NMR data, Table 1 and Table 2 (Figures S29 and S30); 2D NMR data, Figures S31–S34; EIMS *m*/*z* (%) 256 [M]^+^ (<1), 183 (20), 143 (100), 139 (32), 125 (35), 113 (30), 59 (42), 44(100); HREIMS *m*/*z* 256.2034 [M]^+^ (calcd for C_15_H_28_O_3_, 256.2038) (Figure S35).

7,10R-epoxycycloneran-3,15-diol (**6**): Colorless oil; [α]^20^_D_ +6.5 (*c* 0.31, MeOH); IR (KBr) *v*_max_ 3426, 2965, 2931, 2868, 1635, 1460, 1376, 1206, 1152, 1093, 1024, 919 cm^−1^; ^1^H and ^13^C NMR data, Table 1 and Table 2 (Figures S36 and S37); 2D NMR data, Figures S38–S41; EIMS *m*/*z* (%) 256 [M]^+^ (<1), 237 (8), 179 (10), 143 (90), 125 (32), 95 (28), 56 (41), 44(100); HREIMS *m*/*z* 256.2028 [M]^+^ (calcd for C_15_H_28_O_3_, 256.2038) (Figure S42).

(10Z)-15-Acetoxy-10-cycloneren-3,7-diol (**7**): Colorless oil; [α]^20^_D_ − 11 (*c* 0.31, MeOH); IR (KBr) *v*_max_ 3452, 2963, 2932, 1735, 1654, 1460, 1370, 1240, 1025, 920, 885 cm^−1^; ^1^H and ^13^C NMR data, Table 1 and Table 2 (Figures S43 and S44); 2D NMR data, Figures S45–S48; EIMS *m*/*z* (%) 256 [M]^+^ (<1), 139 (33), 125 (98), 107 (28), 81 (27), 44(100); HREIMS *m*/*z* 298.2138 [M]^+^ (calcd for C_17_H_30_O_4_, 298.2144) (Figure S49).

## 4. Conclusions

Chemical examination of the marine-alga-endophytic fungus *Trichoderma asperellum* A-YMD-9-2 led to the isolation and identification of seven new (**1**–**7**) and four known (**8**–**11**) cyclonerane derivatives, including an unprecedented trinorcyclonerane (**4**). The discovery of these new isolates greatly diversifies the unusual cyclonerane sesquiterpenes. Among the isolates, **10** displays significant inhibition of the four phytoplankton species and features no toxicity to any of the zooplankton tested.

## Figures and Tables

**Table 1:** ^1^H NMR Data for **1**–**7** (500 MHz, in CDCl_3_, δ in ppm, *J* in Hz).

Position	1	2	3	4	5	6	7
1 (β)	1.03, d (6.8)	1.03, d (6.8)	1.03, d (6.8)	1.05, d (6.8)	1.02, d (6.8)	1.02, d (6.8)	1.04, d (6.8)
2 (α)	1.56, m	1.55, m	1.59, m	1.60, m	1.50, m	1.54, m	1.60, m
4a	1.67, m	1.69, m	1.67, m	1.69, m	1.68, m	1.68, m	1.68, m
4b	1.56, m	1.56, m	1.55, m	1.56, m	1.59, m	1.58, m	1.55, m
5a	1.88, m	1.88, m	1.85, m	1.88, m	1.89, m	1.89, m	1.85, m
5b	1.63, m	1.56, m	1.54, m	1.55, m	1.49, m	1.40, m	1.55, m
6 (β)	1.97, m	1.89, m	1.85, m	1.87, m	1.95, m	2.00, m	1.84, m
8a	2.12, m	1.73, m	1.44, m	1.57, m	1.75, m	1.86, m	1.50, t (8.3)
8b	1.89, m	1.59, m			1.64, m	1.69, m	
9a	2.51, m	1.61, m	1.44, m	1.68, m	1.92, m	1.83, m	2.16, m
9b		1.40, m			1.71, m	1.79, m	
10		3.38, br d (10.3) ^a^	1.45, m	3.68, m	4.07, ddd (7.6, 6.9, 4.1)	4.04, ddd (9.8, 5.6, 4.3)	5.41, t (7.3)
11					1.93, m	2.00, m	
12	1.30, s	1.16, s	1.22, s		0.93, d (7.0)	0.90, d (7.1)	1.74, br s
13 (α)	1.25, s	1.25, s	1.25, s	1.26, s	1.24, s	1.25, s	1.25, s
14	1.19, s	1.15, s	1.16, s	1.17, s	1.14, s	1.17, s	1.16, s
15a	1.29, s	1.21, s	1.22, s		3.65, d (10.8, 6.1)	3.69, dd (10.8, 6.9)	4.62, d (11.9)
15b					3.61, d (10.8, 4.1)	3.58, dd (10.8, 3.7)	4.57, d (11.9)
CH_3_CO							2.06, s

^a^ H-10 is coupled with H-9a and H-9b, but one of the couplings only results in broad resonance.

**Table 2:** ^13^C NMR data for **1**–**7** (125 MHz, in CDCl_3_, δ in ppm).

Position	δ_C_, Type						
	1	2	3	4	5	6	7
1	14.4, CH_3_	14.5, CH_3_	14.7, CH_3_	14.6, CH_3_	14.0, CH_3_	13.8, CH_3_	14.7, CH_3_
2	44.9, CH	44.7, CH	44.4, CH	44.5, CH	45.4, CH	45.3, CH	44.4, CH
3	81.4, C	81.5, C	81.5, C	81.4, C	81.3, C	81.4, C	81.4, C
4	40.4, CH_2_	40.4, CH_2_	40.5, CH_2_	40.5, CH_2_	40.5, CH_2_	40.4, CH_2_	40.5, CH_2_
5	24.8, CH_2_	24.5, CH_2_	24.4, CH_2_	24.6, CH_2_	25.1, CH_2_	25.4, CH_2_	24.5, CH_2_
6	55.1, CH	54.5, CH	54.3, CH	54.9, CH	54.2, CH	54.1, CH	54.6, CH
7	76.0, C	75.1, C	75.0, C	74.8, C	85.7, C	86.0, C	74.8, C
8	31.5, CH_2_	37.0, CH_2_	41.1, CH_2_	36.8, CH_2_	35.7, CH_2_	34.8, CH_2_	40.5, CH_2_
9	32.8, CH_2_	25.8, CH_2_	18.7, CH_2_	27.2, CH_2_	28.1, CH_2_	27.4, CH_2_	22.5, CH_2_
10	215.1, C	79.0, CH	44.5, CH_2_	63.6, CH_2_	80.3, CH	84.2, CH	131.1, CH
11	79.4, C	73.4, C	71.2, C		38.2, CH	37.4, CH	130.0, C
12	27.8, CH_3_	23.5, CH_3_	29.4, CH_3_		11.8, CH_3_	12.0, CH_3_	21.6, CH_3_
13	26.3, CH_3_	26.2, CH_3_	26.2, CH_3_	26.2, CH_3_	26.2, CH_3_	26.2, CH_3_	26.2, CH_3_
14	23.5, CH_3_	25.0, CH_3_	25.2, CH_3_	25.2, CH_3_	23.1, CH_3_	26.4, CH_3_	25.0, CH_3_
15	28.0, CH_3_	26.7, CH_3_	29.5, CH_3_		66.9, CH_2_	66.7, CH_2_	63.3, CH_2_
CH_3_CO							171.3, C
CH_3_CO							21.1, CH_3_

**Table 3:** Inhibition against four marine phytoplankton species by **1**–**11**.

Compound	IC_50_ (μg/mL)			
	*Chattonella marina*	*Heterosigma akashiwo*	*Karlodinium veneficum*	*Prorocentrum donghaiense*
**1**	5.2	8.0	10	9.9
**2**	8.8	21	76	6.5
**3**	61	73	71	40
**4**	13	73	6.3	34
**5**	2.4	26	3.9	20
**6**	5.8	37	5.5	15
**7**	59	14	35	7.3
**8**	42	50	12	3.4
**9**	15	53	43	1.1
**10**	1.6	1.8	1.6	2.0
**11**	62	9.4	13	6.0

## Supplementary Materials

The following are available online at https://www.mdpi.com/1660-3397/17/5/252/s1, Figures S1–S49: 1D/2D NMR and HRMS spectra of compounds **1**–**7**.
