# Supplementary material for: Cyclonerane Derivatives from the Algicolous Endophytic Fungus Trichoderma asperellum A-YMD-9-2
Source: Mar Drugs. 2019 Apr 28;17(5):252. doi: 10.3390/md17050252 (PMC6562392; doi:10.3390/md17050252)
Supplement: Supplementary file 1 [file marinedrugs-17-00252-s001.pdf]

Supplementary Materials

Cyclonerane Derivatives from the Algicolous Endophytic Fungus *Trichoderma asperellum* A-YMD-9-2

Yin-Ping Song [1,2,3], Feng-Ping Miao [1,3], Xiang-Hong Liu [1,2,3], Xiu-Li Yin [1,3] and Nai-Yun Ji [1,3,*]

[1] Yantai Institute of Coastal Zone Research, Chinese Academy of Sciences, Yantai 264003, People's Republic of China; ypsong@yic.ac.cn (Y.-P.S.); fpmiao@yic.ac.cn (F.-P.M.); xianghongliu@yic.ac.cn (X.-H.L.); xlyin@yic.ac.cn@yic.ac.cn (X.-L.Y.)

[2] University of Chinese Academy of Sciences, Beijing 100049, People's Republic of China

[3] Center for Ocean Mega-Science, Chinese Academy of Sciences, Qingdao 266071, People's Republic of China

* Correspondence: nyji@yic.ac.cn; Tel.: +86-535-210-9176

Contents

Figure S1. ^{1}H NMR spectrum of compound **1** in $CDCl_3$.

Figure S2. ^{13}C NMR and DEPT spectra of compound **1** in $CDCl_3$.

Figure S3. HSQC spectrum of compound **1** in $CDCl_3$.

Figure S4. HMBC spectrum of compound **1** in $CDCl_3$.

Figure S5. COSY spectrum of compound **1** in $CDCl_3$.

Figure S6. NOESY spectrum of compound **1** in $CDCl_3$.

Figure S7. HREIMS spectrum of compound **1**.

Figure S8. ^{1}H NMR spectrum of compound **2** in $CDCl_3$.

Figure S9. ^{13}C NMR and DEPT spectra of compound **2** in $CDCl_3$.

Figure S10. HSQC spectrum of compound **2** in $CDCl_3$.

Figure S11. HMBC spectrum of compound **2** in $CDCl_3$.

Figure S12. COSY spectrum of compound **2** in $CDCl_3$.

Figure S13. NOESY spectrum of compound **2** in $CDCl_3$.

Figure S14. HRESI^{+}MS spectrum of compound **2**.

Figure S15. ^{1}H NMR spectrum of compound **3** in $CDCl_3$.

Figure S16. ^{13}C NMR and DEPT spectra of compound **3** in $CDCl_3$.

Figure S17. HSQC spectrum of compound **3** in $CDCl_3$.

Figure S18. HMBC spectrum of compound **3** in $CDCl_3$.

Figure S19. COSY spectrum of compound **3** in $CDCl_3$.

Figure S20. NOESY spectrum of compound **3** in $CDCl_3$.

Figure S21. HREIMS spectrum of compound **3**.

Figure S22. ^{1}H NMR spectrum of compound **4** in $CDCl_3$.

Figure S23. ^{13}C NMR and DEPT spectra of compound **4** in $CDCl_3$.

Figure S24. HSQC spectrum of compound **4** in $CDCl_3$.

Figure S25. HMBC spectrum of compound **4** in $CDCl_3$.

Figure S26. COSY spectrum of compound **4** in $CDCl_3$.

Figure S27. NOESY spectrum of compound **4** in $CDCl_3$.

Figure S28. HREIMS spectrum of compound **4**.

Figure S29. ^{1}H NMR spectrum of compound **5** in $CDCl_3$.

Figure S30. ^{13}C NMR and DEPT spectra of compound **5** in $CDCl_3$.

Figure S31. HSQC spectrum of compound **5** in $CDCl_3$.

Figure S32. HMBC spectrum of compound **5** in $CDCl_3$.

Figure S33. COSY spectrum of compound **5** in $CDCl_3$.

Figure S34. NOESY spectrum of compound **5** in $CDCl_3$.

Figure S35. HREIMS spectrum of compound **5**.

Figure S36. ^{1}H NMR spectrum of compound **6** in $CDCl_3$.

Figure S37. ^{13}C NMR and DEPT spectra of compound **6** in $CDCl_3$.

Figure S38. HSQC spectrum of compound **6** in $CDCl_3$.

Figure S39. HMBC spectrum of compound **6** in $CDCl_3$.

Figure S40. COSY spectrum of compound **6** in $CDCl_3$.

Figure S41. NOESY spectrum of compound **6** in $CDCl_3$.

Figure S42. HREIMS spectrum of compound **6**.

Figure S43. ^{1}H NMR spectrum of compound **7** in $CDCl_3$.

Figure S44. ^{13}C NMR and DEPT spectra of compound **7** in $CDCl_3$.

Figure S45. HSQC spectrum of compound **7** in $CDCl_3$.

Figure S46. HMBC spectrum of compound **7** in $CDCl_3$.

Figure S47. COSY spectrum of compound **7** in $CDCl_3$.

Figure S48. NOESY spectrum of compound **7** in $CDCl_3$.

Figure S49. HREIMS spectrum of compound **7**.

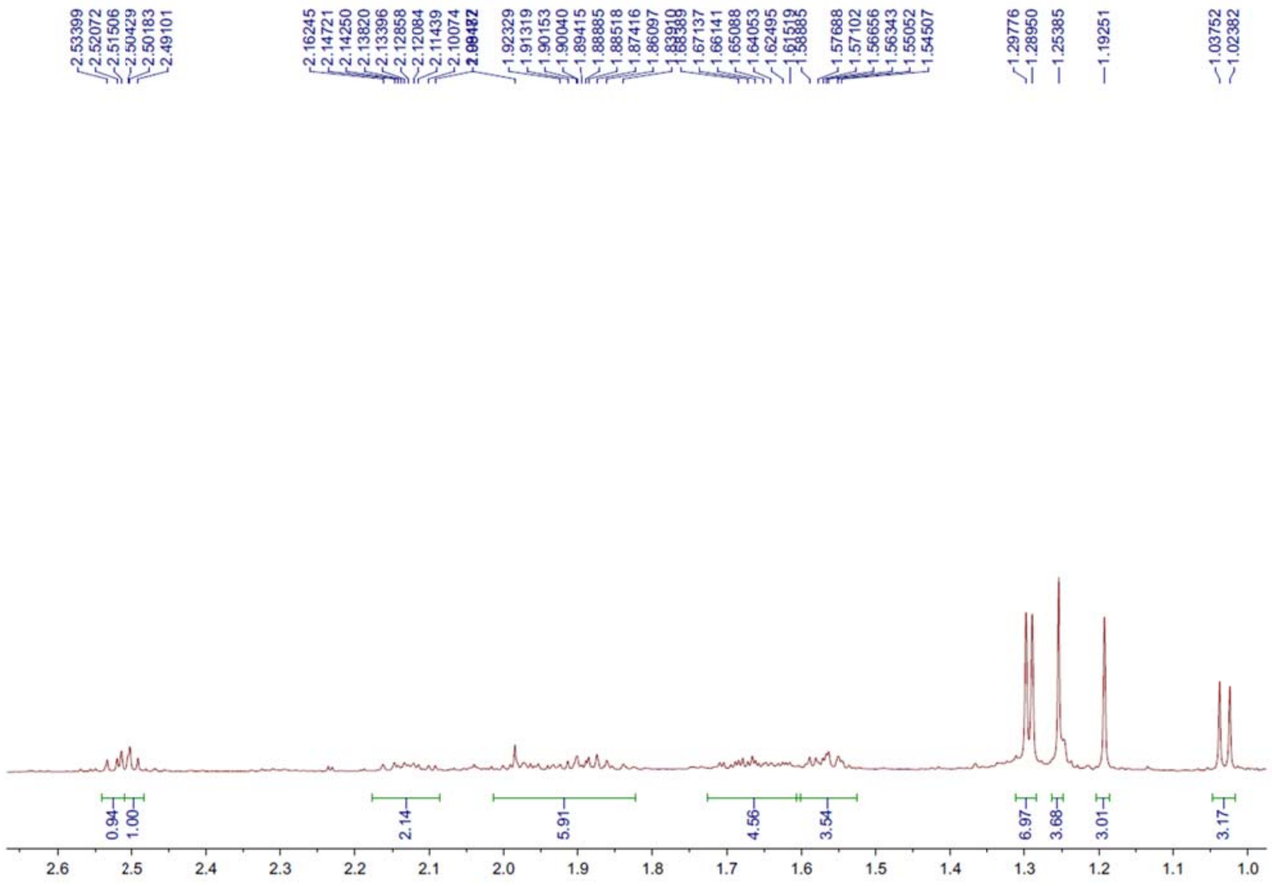

Figure S1. ^{1}H NMR spectrum of compound **1** in $CDCl_3$.

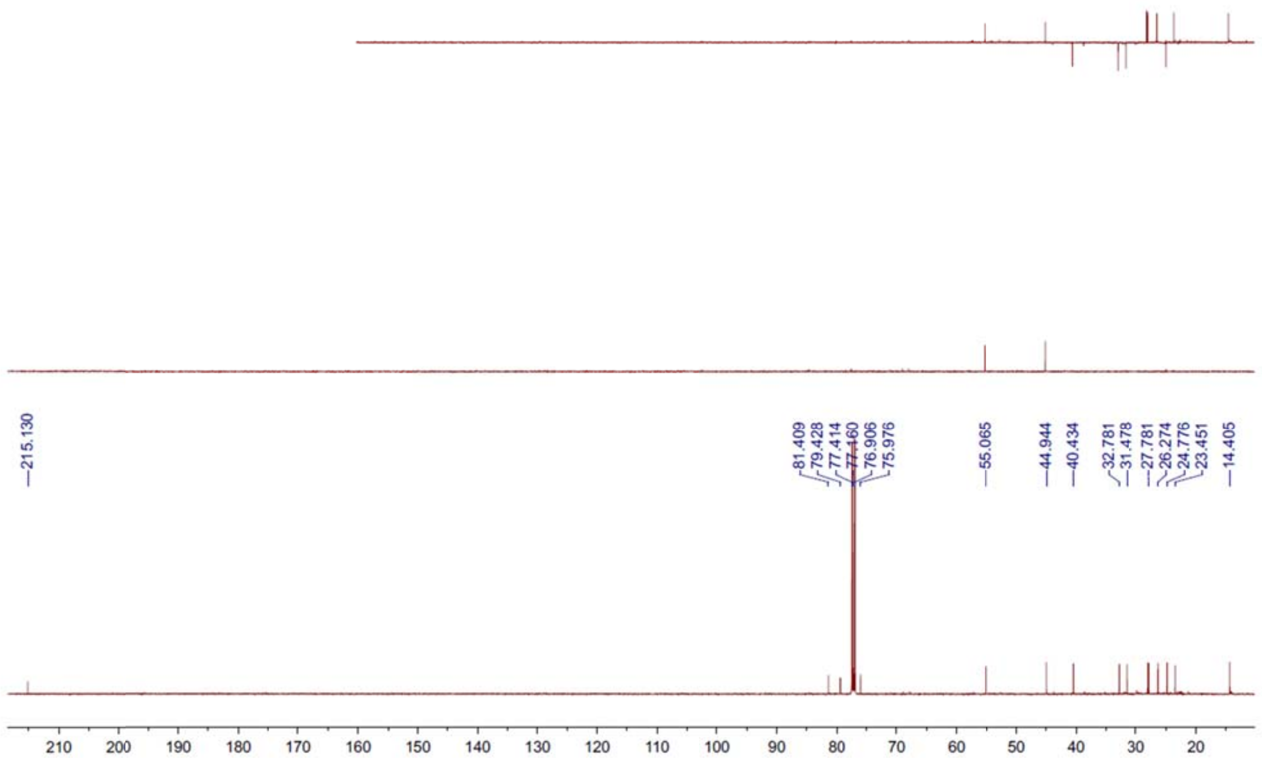

Figure S2. ^{13}C NMR and DEPT spectra of compound **1** in $CDCl_3$.

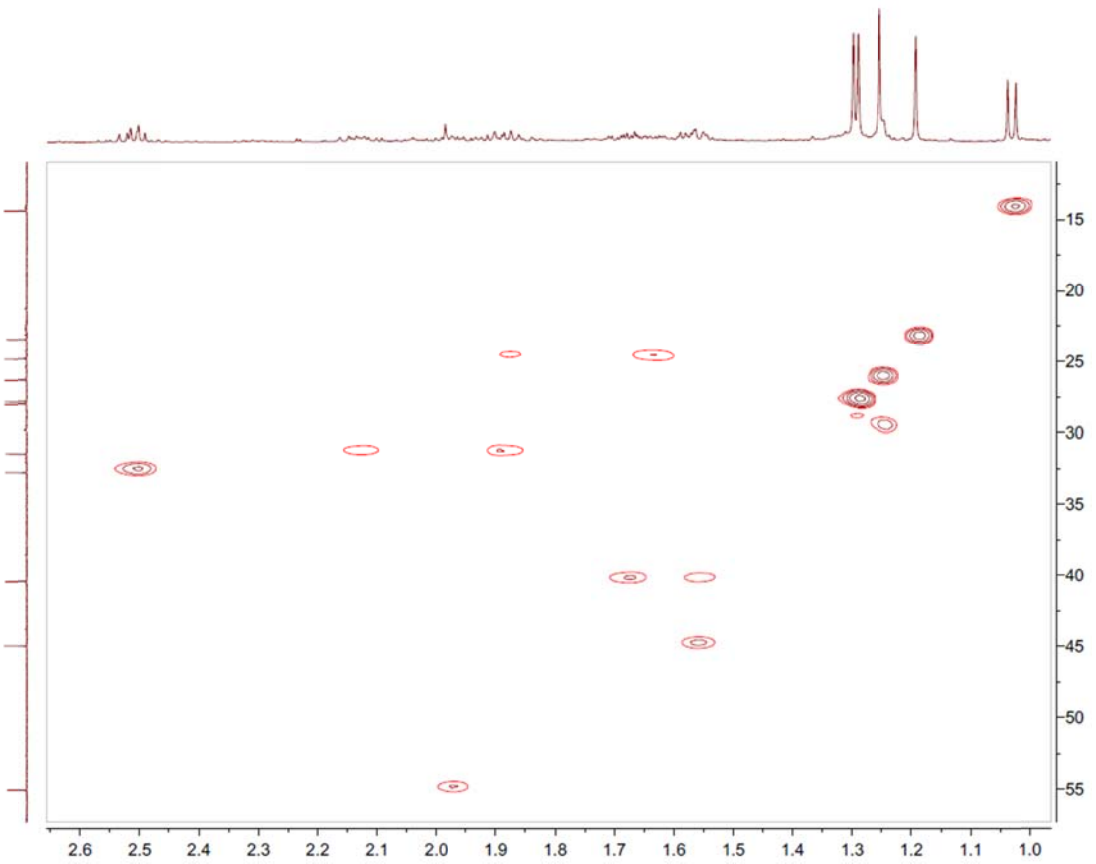

Figure S3. HSQC spectrum of compound **1** in $CDCl_3$.

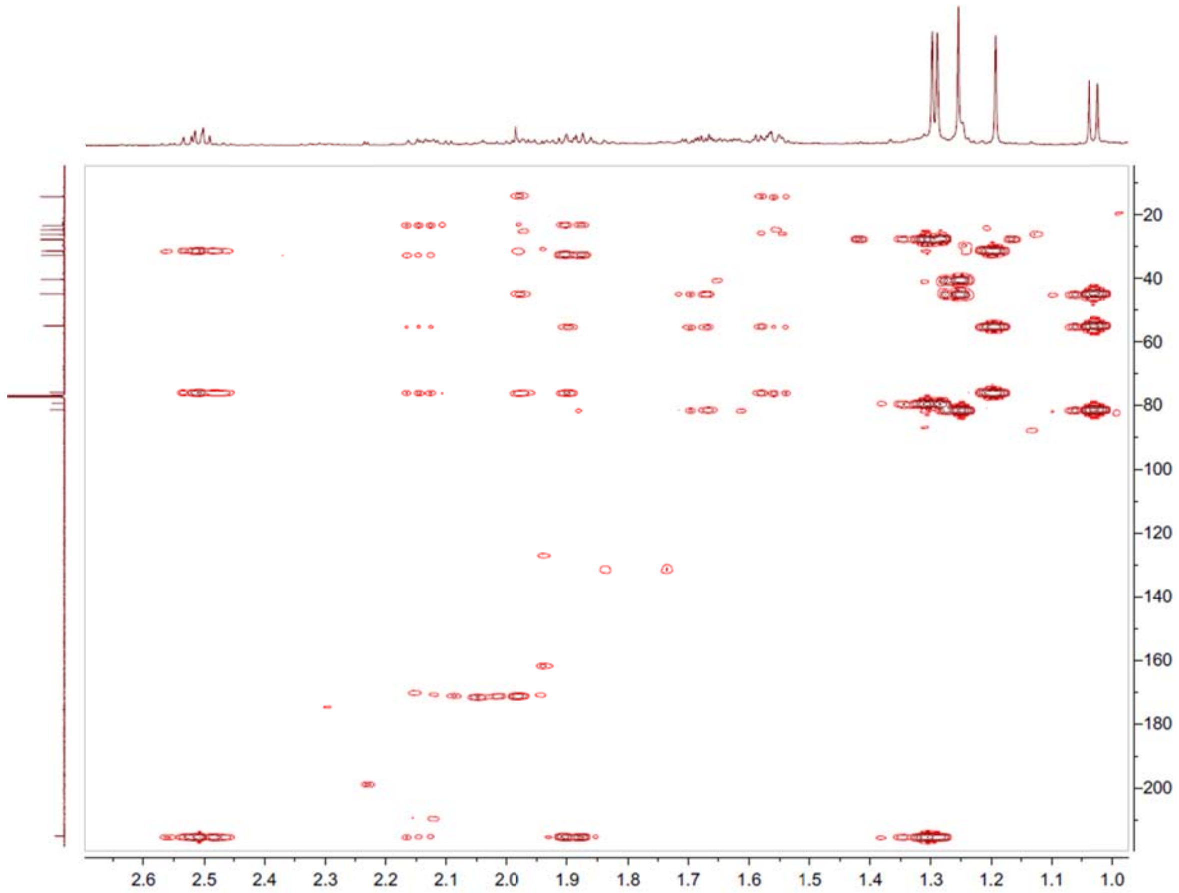

Figure S4. HMBC spectrum of compound **1** in $CDCl_3$.

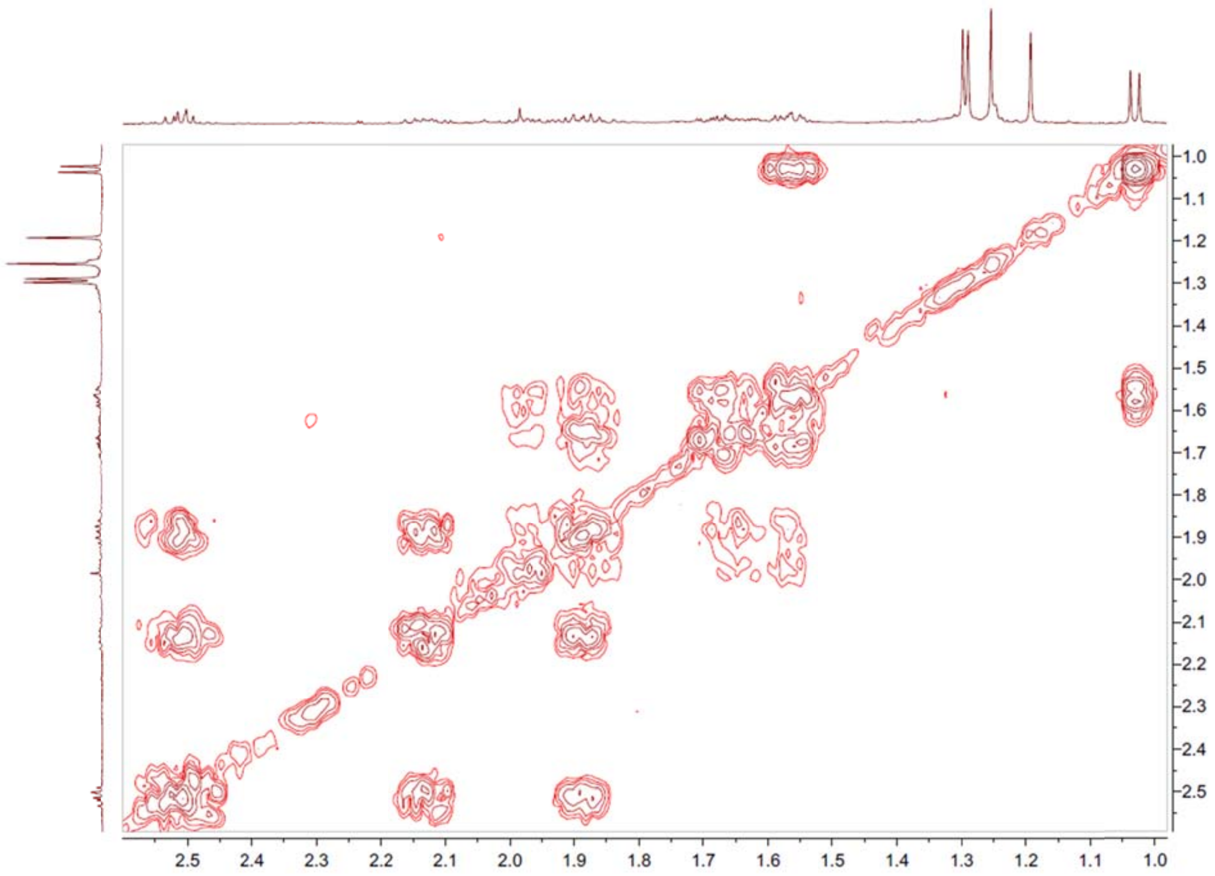

Figure S5. COSY spectrum of compound **1** in $CDCl_3$.

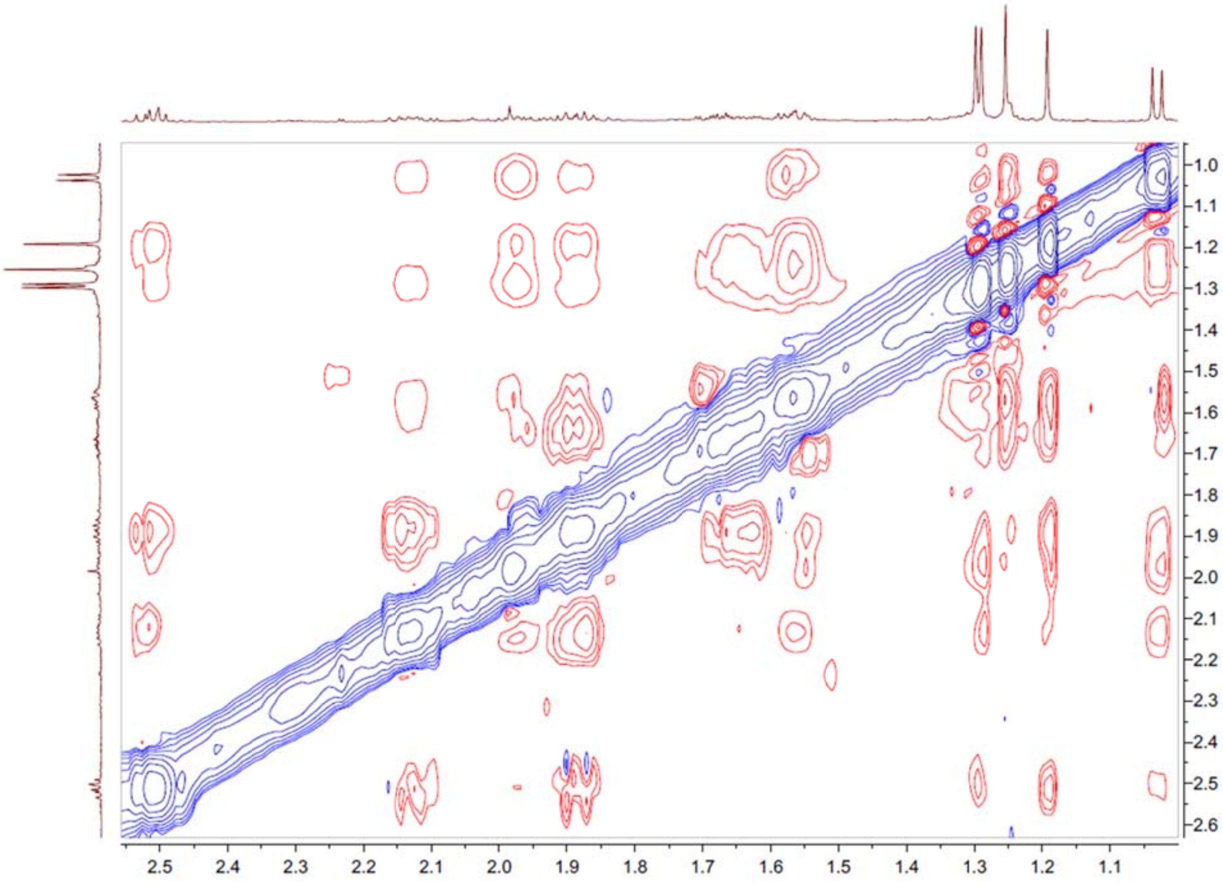

Figure S6. NOESY spectrum of compound **1** in $CDCl_3$**.**

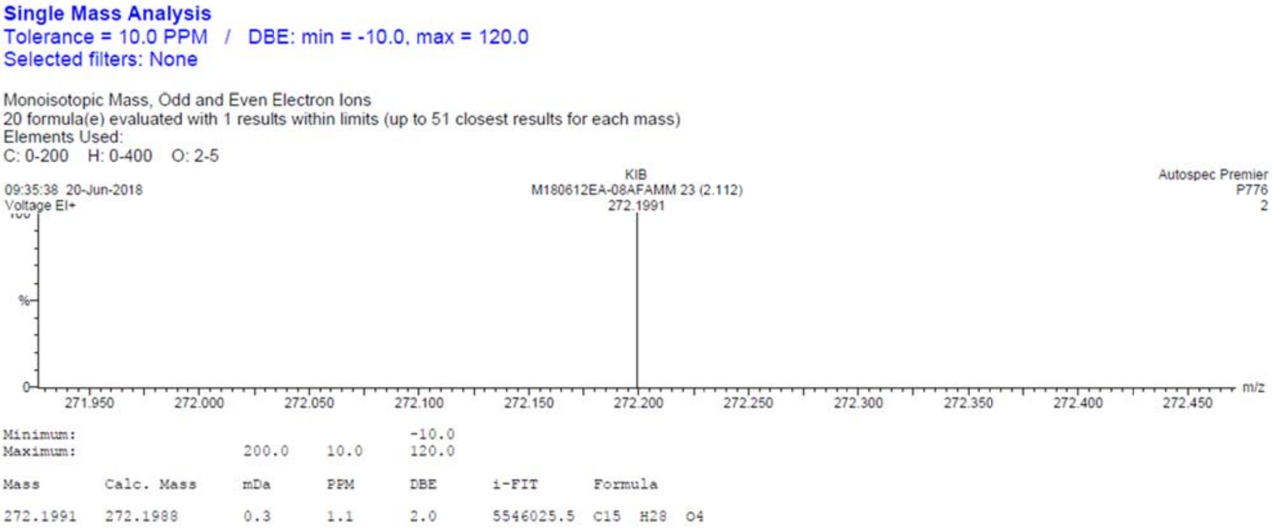

Figure S7. HREIMS spectrum of compound **1**.

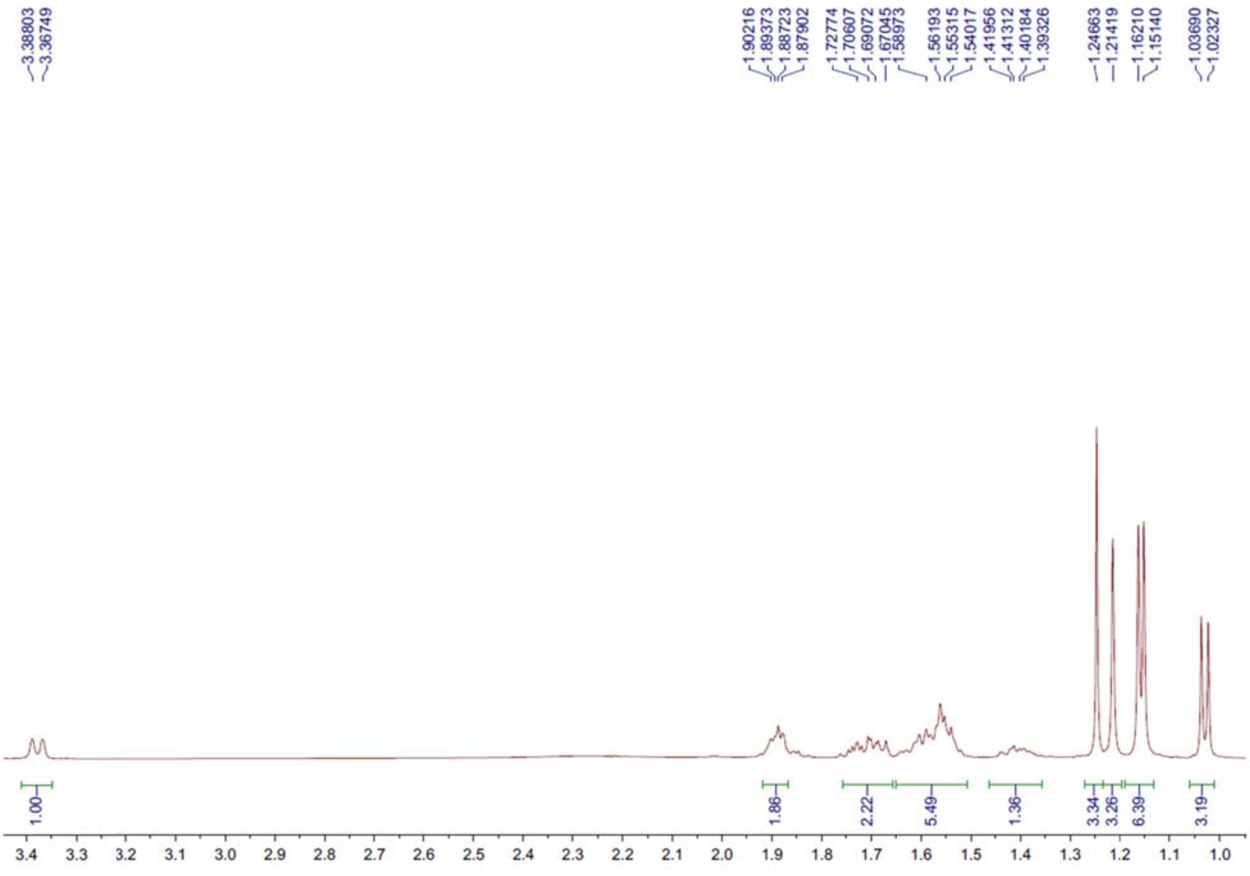

Figure S8. ^{1}H NMR spectrum of compound **2** in $CDCl_3$.

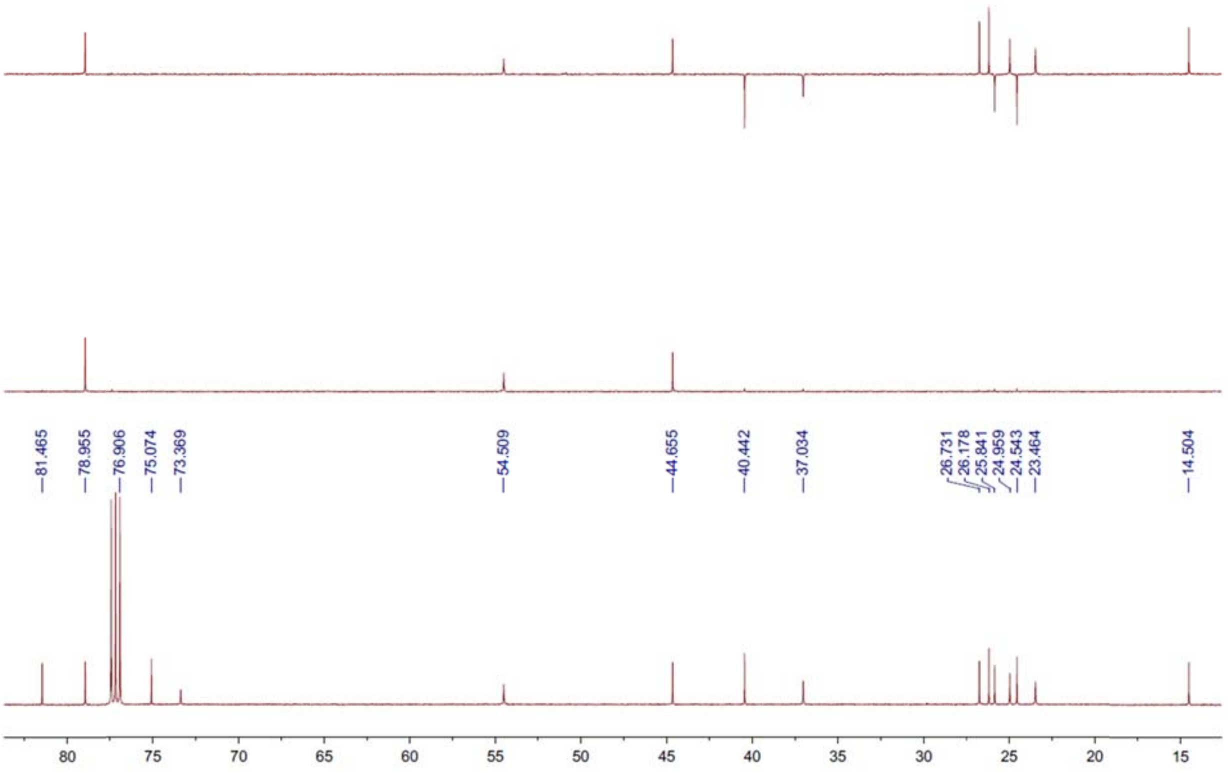

Figure S9. ^{13}C NMR and DEPT spectra of compound **2** in $CDCl_3$.

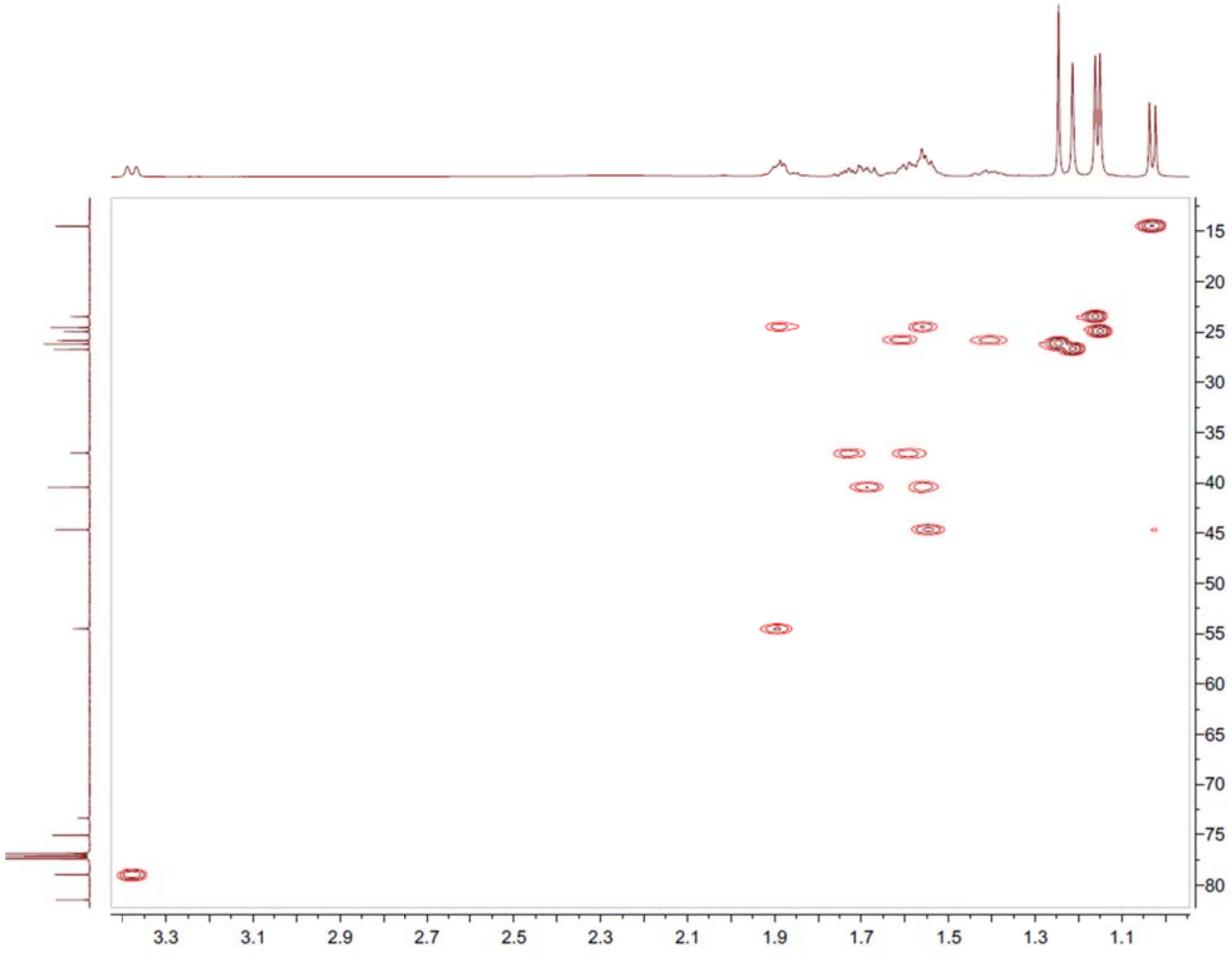

Figure S10. HSQC spectrum of compound **2** in $CDCl_3$.

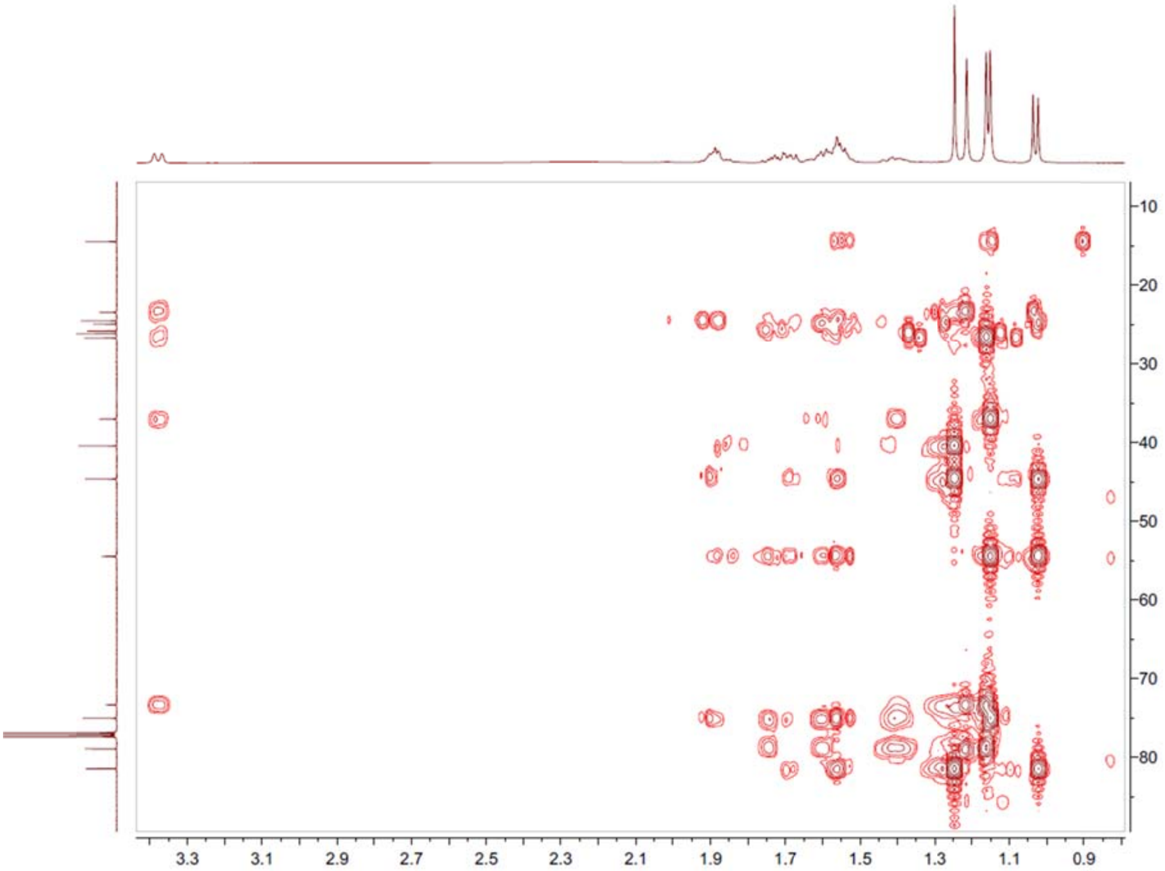

Figure S11. HMBC spectrum of compound **2** in $CDCl_3$.

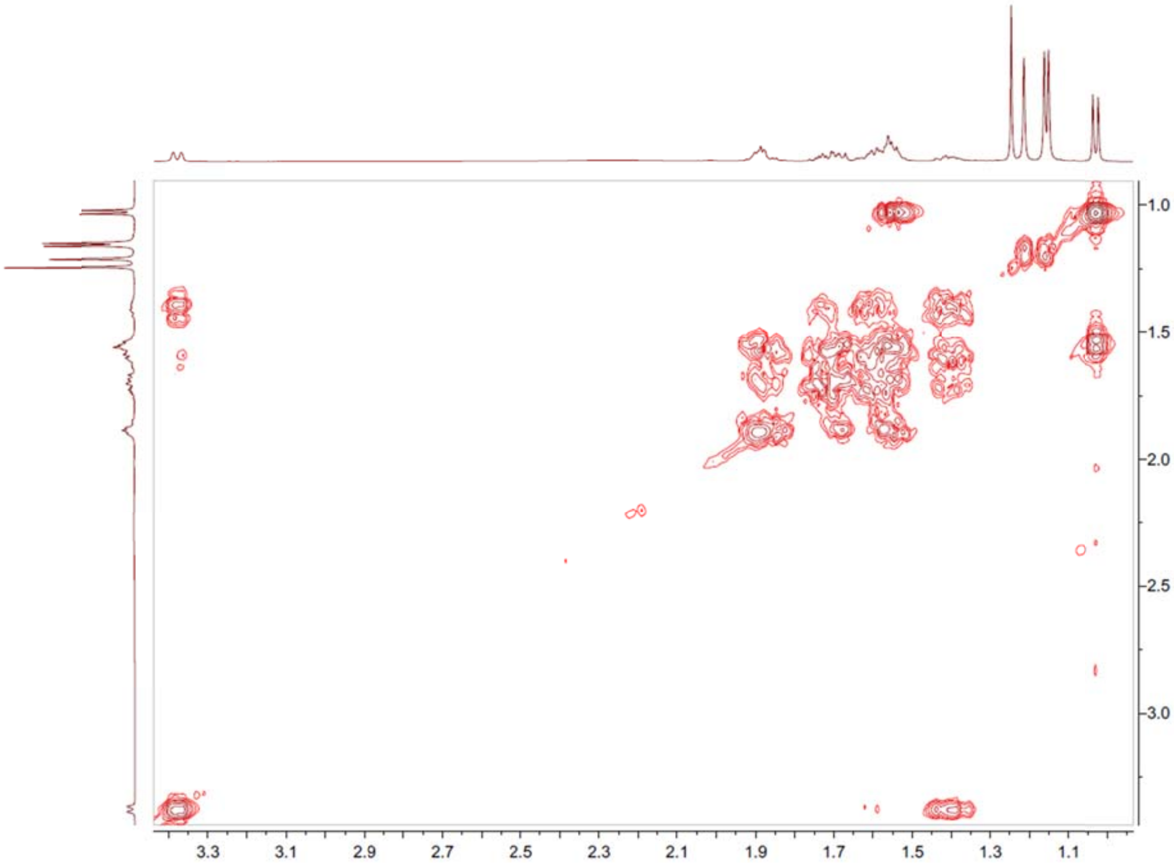

Figure S12. COSY spectrum of compound **2** in $CDCl_3$.

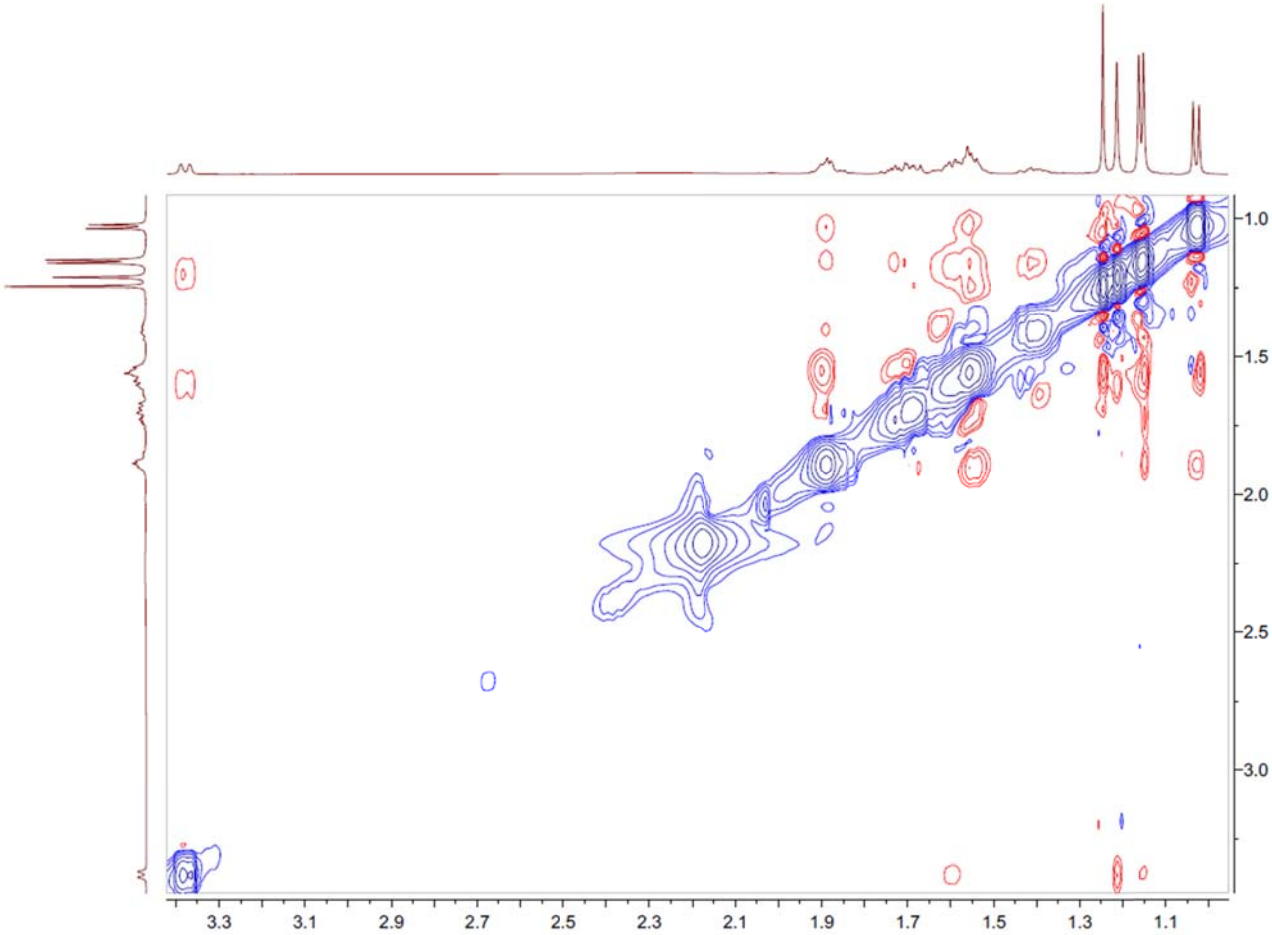

Figure S13. NOESY spectrum of compound **2** in $CDCl_3$.

Qualitative Analysis Report

**Data Filename**	181121ESIA4.d	**Sample Name**	compound **2**
**Sample Type**	Sample	**Position**	
**Instrument Name**	Agilent G6230 TOF MS	**User Name**	KIB
**Acq Method**	ESI.m	**Acquired Time**	11/21/2018 9:39:20 AM
**IRM Calibration Status**	Success	**DA Method**	ESI.m
**Comment**			

Sample Group **Info.**
Acquisition SW 6200 series TOF/6500 series
Version Q-TOF B.05.01 (B5125.2)

User Spectra

Fragmentor Voltage	Collision Energy	Ionization Mode
200	0	ESI

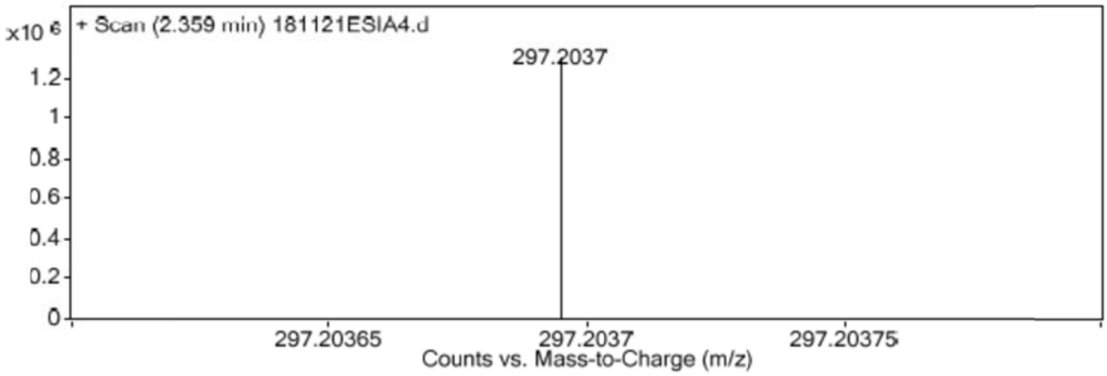

Peak List

m/z	z	Abund	Formula	Ion
297.2037	1	1295062.75	C15 H30 Na O4	M+
298.2073	1	196830.22	C15 H30 Na O4	M+
310.1265	1	172631.41		
322.1268	1	651617.13		
338.2304	1	159140.7		
344.1088	1	166444.25		
571.4188	1	1124473.88		
572.4223	1	360450.06		
575.356	1	156204.05		
687.4812	1	189309.59		

Formula Calculator Element Limits

Element	Min	Max
C	0	200
H	0	400
O	0	10
Na	1	1

Formula Calculator Results

Formula	CalculatedMass	Mz	Diff.(mDa)	Diff. (ppm)	DBE
C15 H30 Na O4	297.2042	297.2037	0.5	1.6	0.5

--- End Of Report ---

Figure S14. HRESI$^+$MS spectrum of compound **2**.

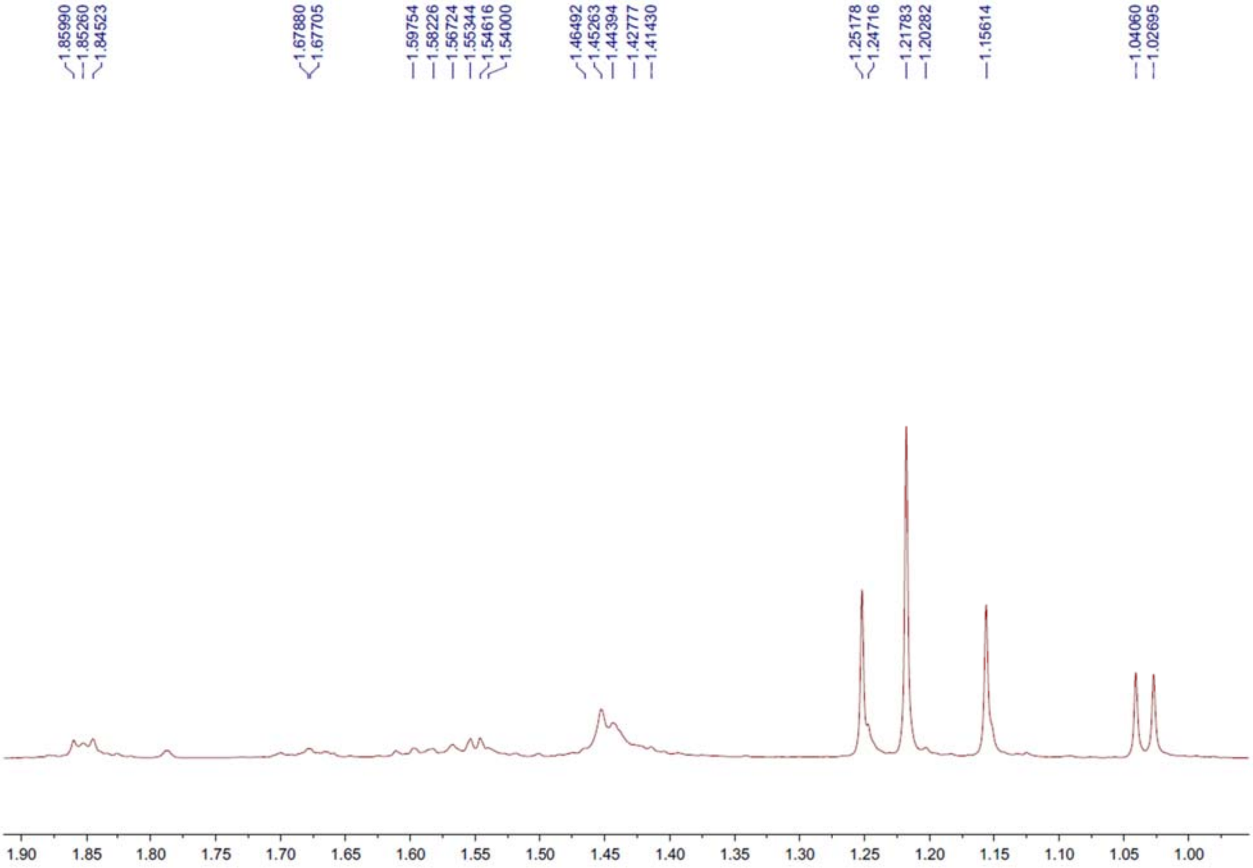

Figure S15. ^{1}H NMR spectrum of compound **3** in $CDCl_3$.

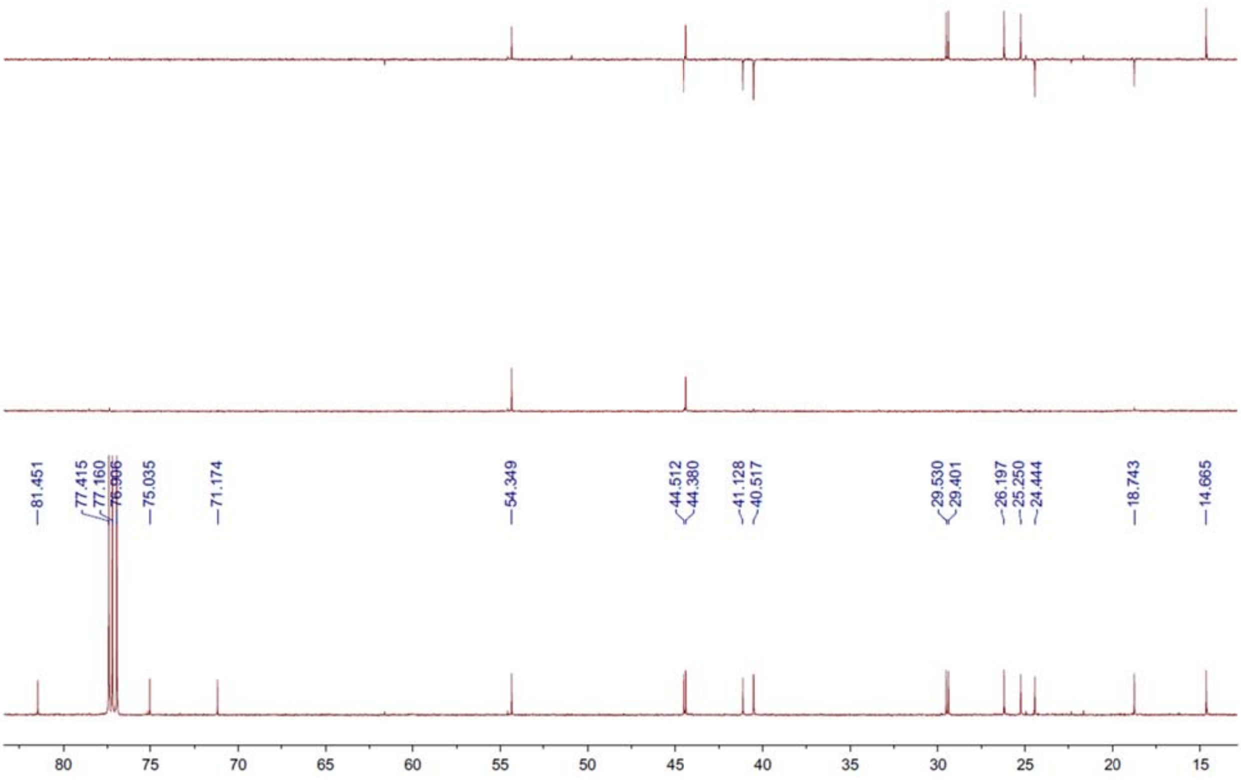

Figure S16. ^{13}C NMR and DEPT spectra of compound **3** in $CDCl_3$.

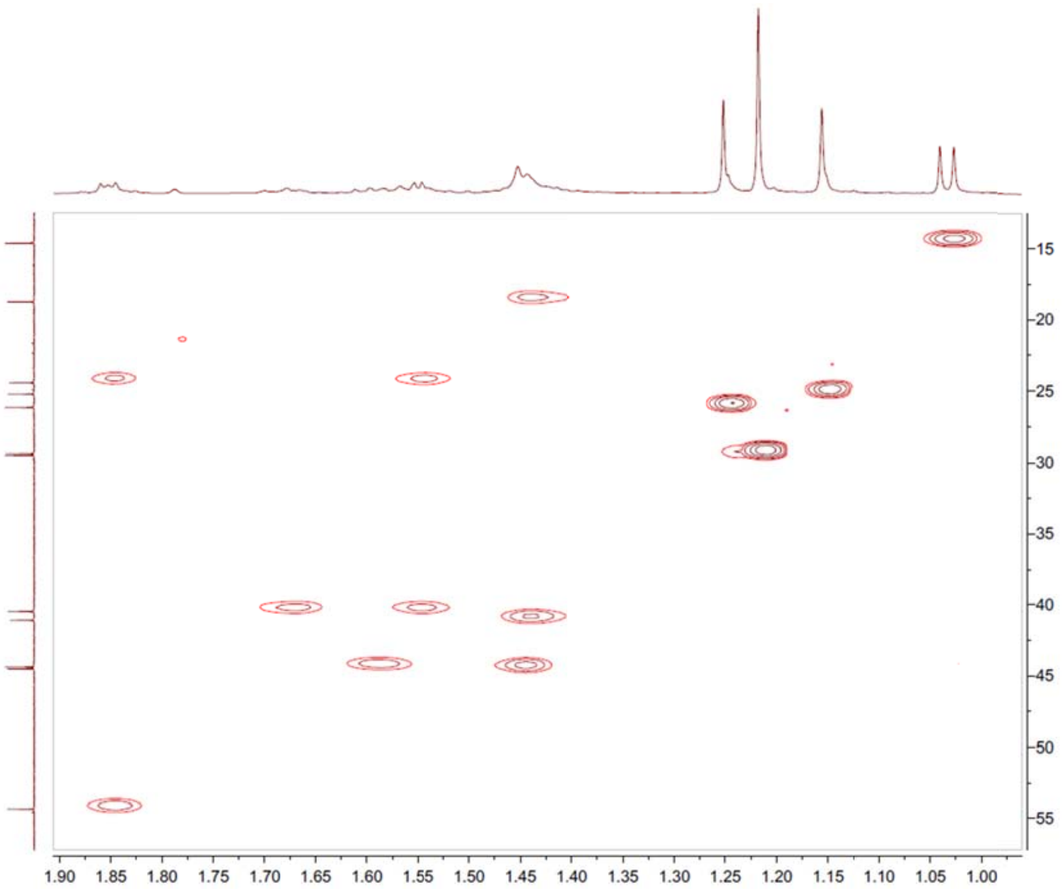

Figure S17. HSQC spectrum of compound **3** in $CDCl_3$.

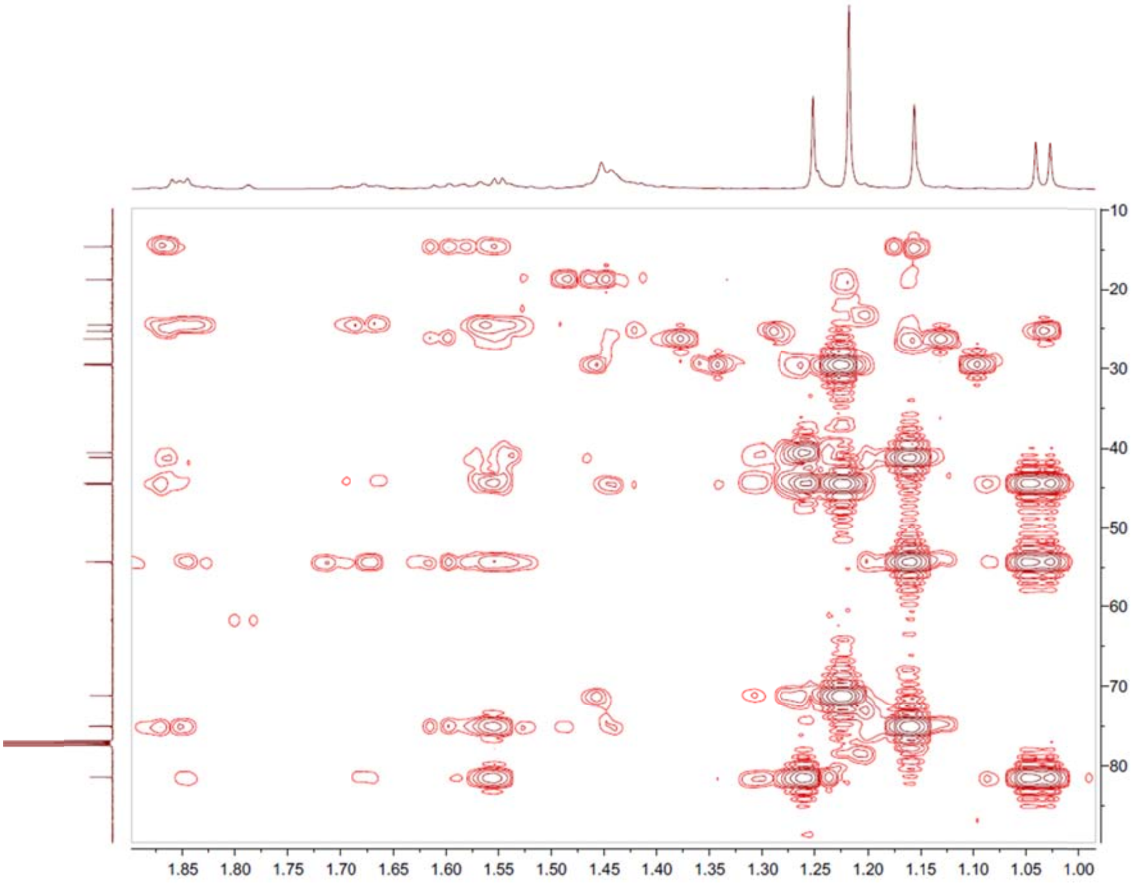

Figure S18. HMBC spectrum of compound **3** in $CDCl_3$.

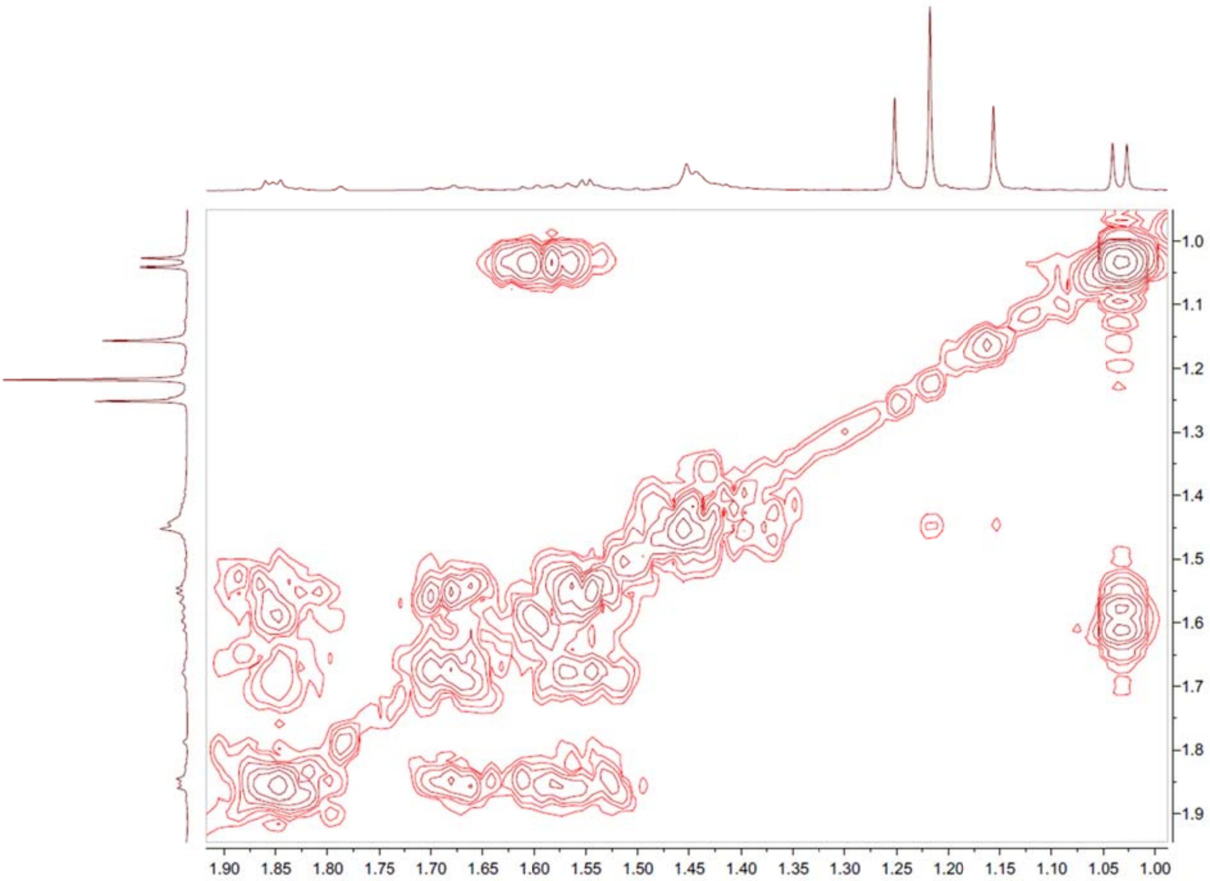

Figure S19. COSY spectrum of compound **3** in $CDCl_3$.

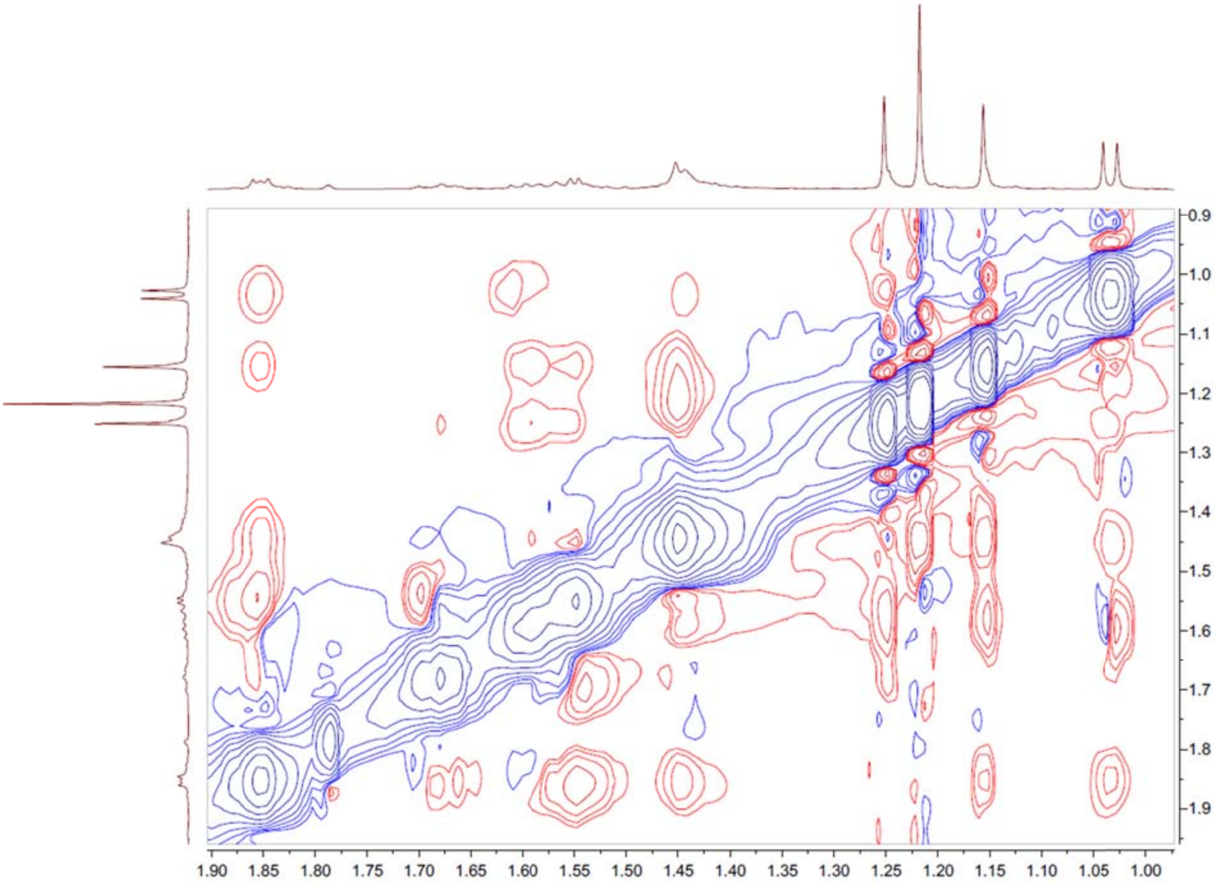

Figure S20. NOESY spectrum of compound **3** in $CDCl_3$.

Single Mass Analysis
Tolerance = 10.0 PPM / DBE: min = -10.0, max = 120.0
Selected filters: None

Monoisotopic Mass, Odd and Even Electron Ions
22 formula(e) evaluated with 1 results within limits (up to 51 closest results for each mass)
Elements Used:
C: 0-200 H: 0-400 O: 2-6

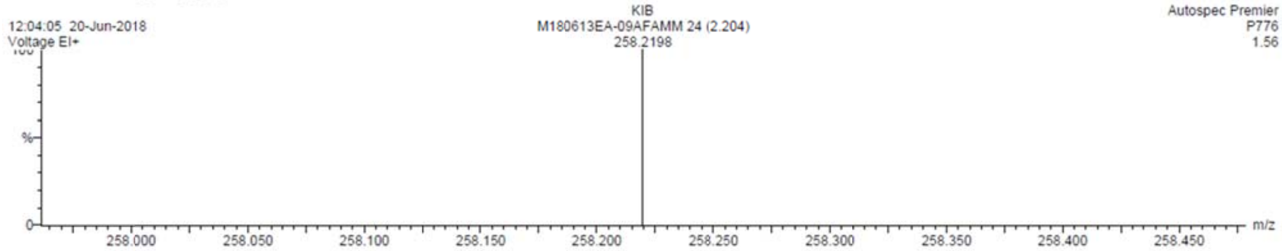

Minimum:				-10.0		
Maximum:		200.0	10.0	120.0		
Mass	Calc. Mass	mDa	PPM	DBE	i-FIT	Formula
258.2198	258.2195	0.3	1.2	1.0	5546025.5	C15 H30 O3

Figure S21. HREIMS spectrum of compound **3**.

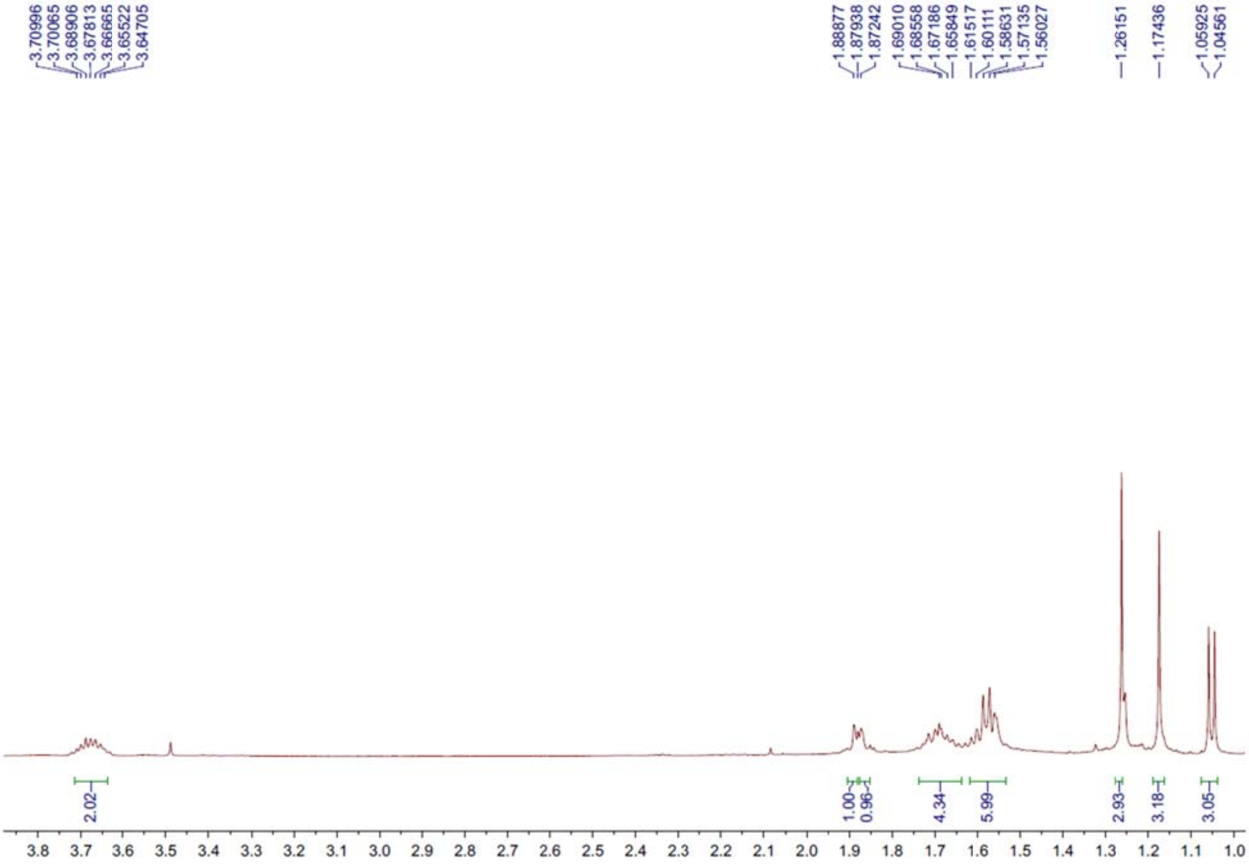

Figure S22. ^{1}H NMR spectrum of compound **4** in $CDCl_3$.

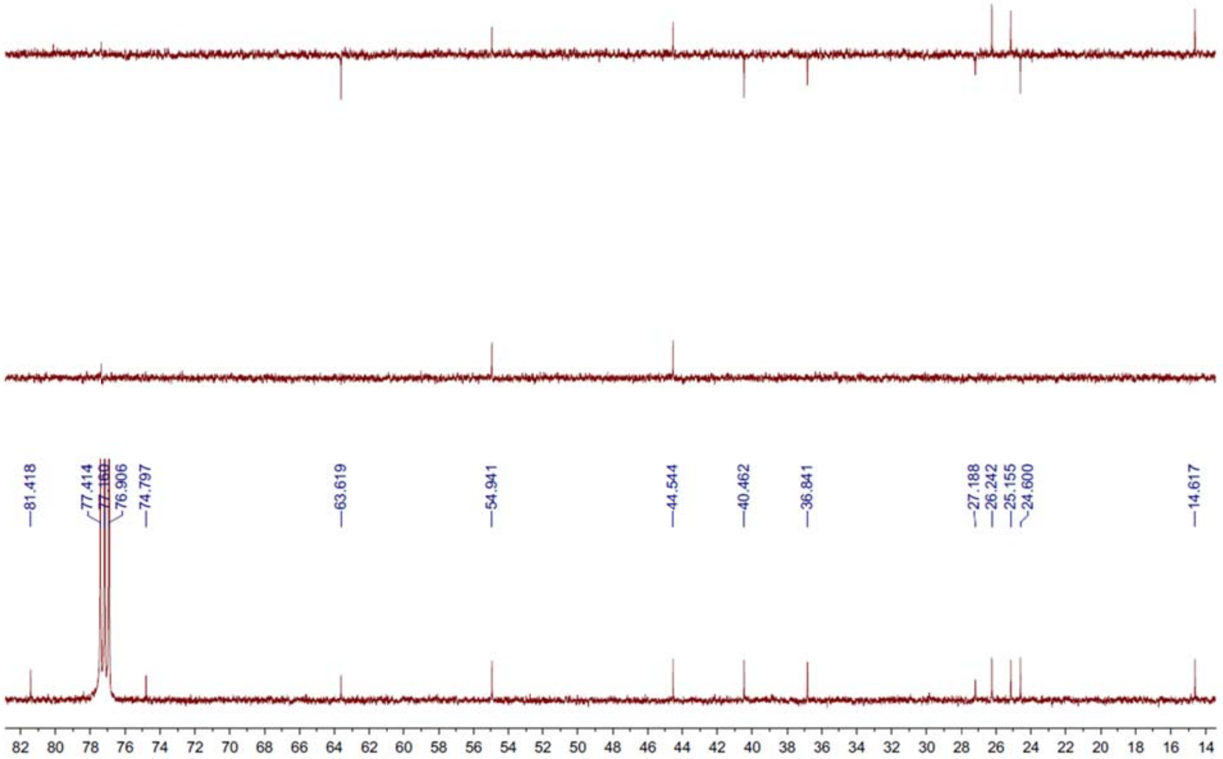

Figure S23. ^{13}C NMR and DEPT spectra of compound **4** in $CDCl_3$.

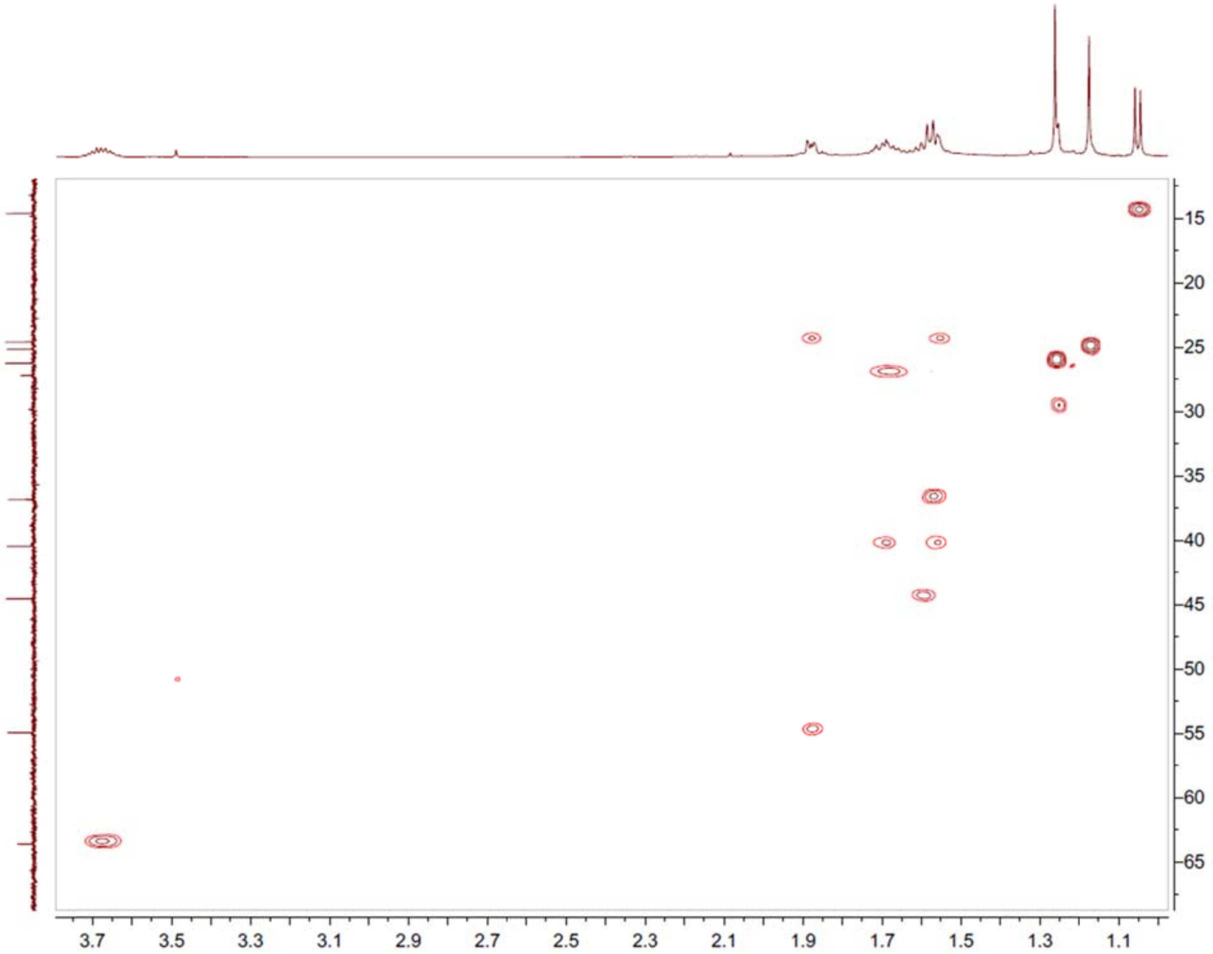

Figure S24. HSQC spectrum of compound **4** in $CDCl_3$.

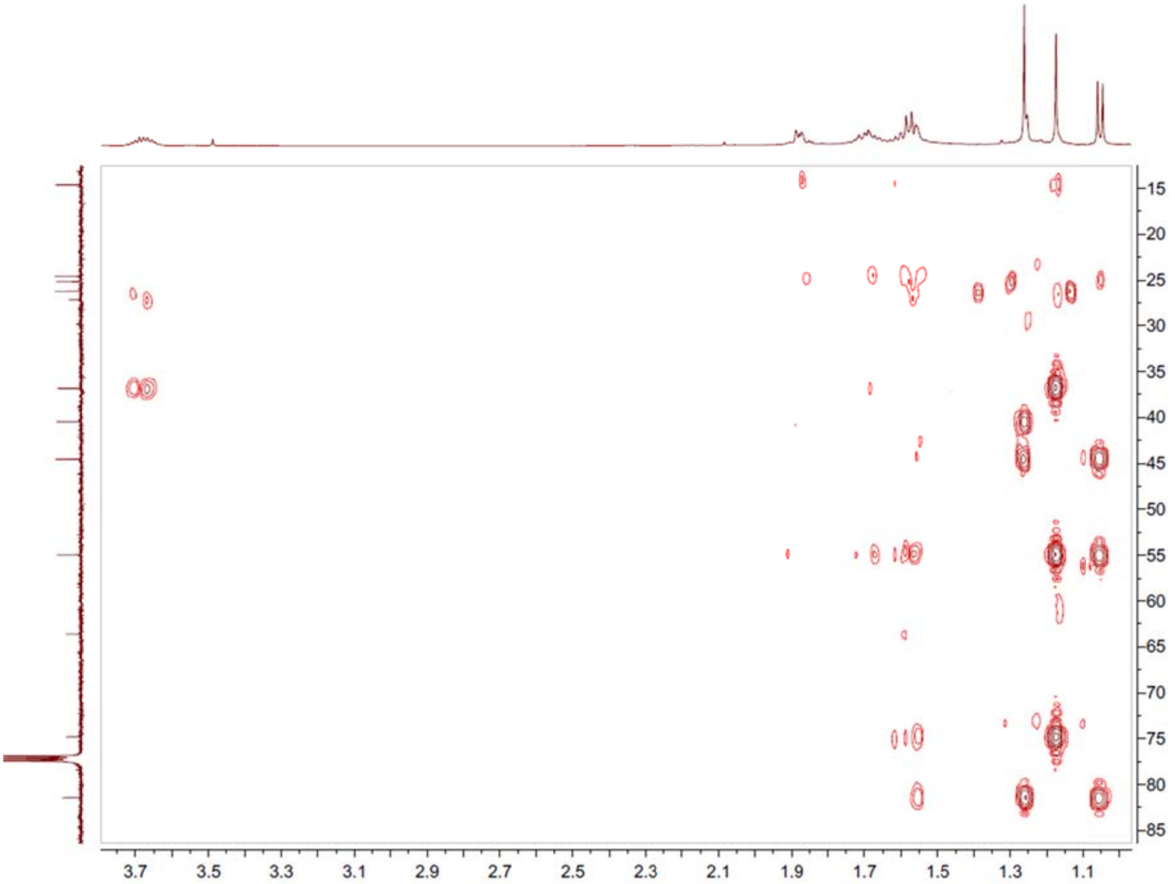

Figure S25. HMBC spectrum of compound **4** in $CDCl_3$.

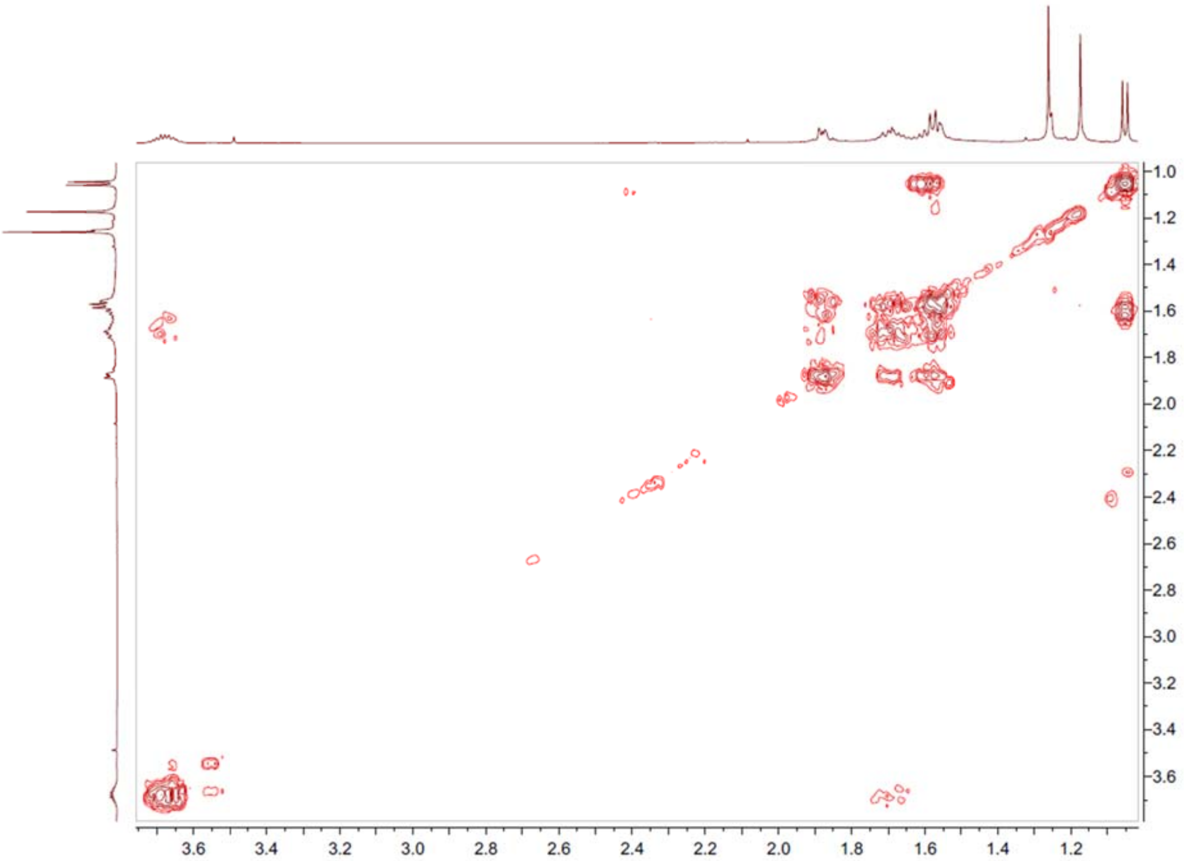

Figure S26. COSY spectrum of compound **4** in $CDCl_3$.

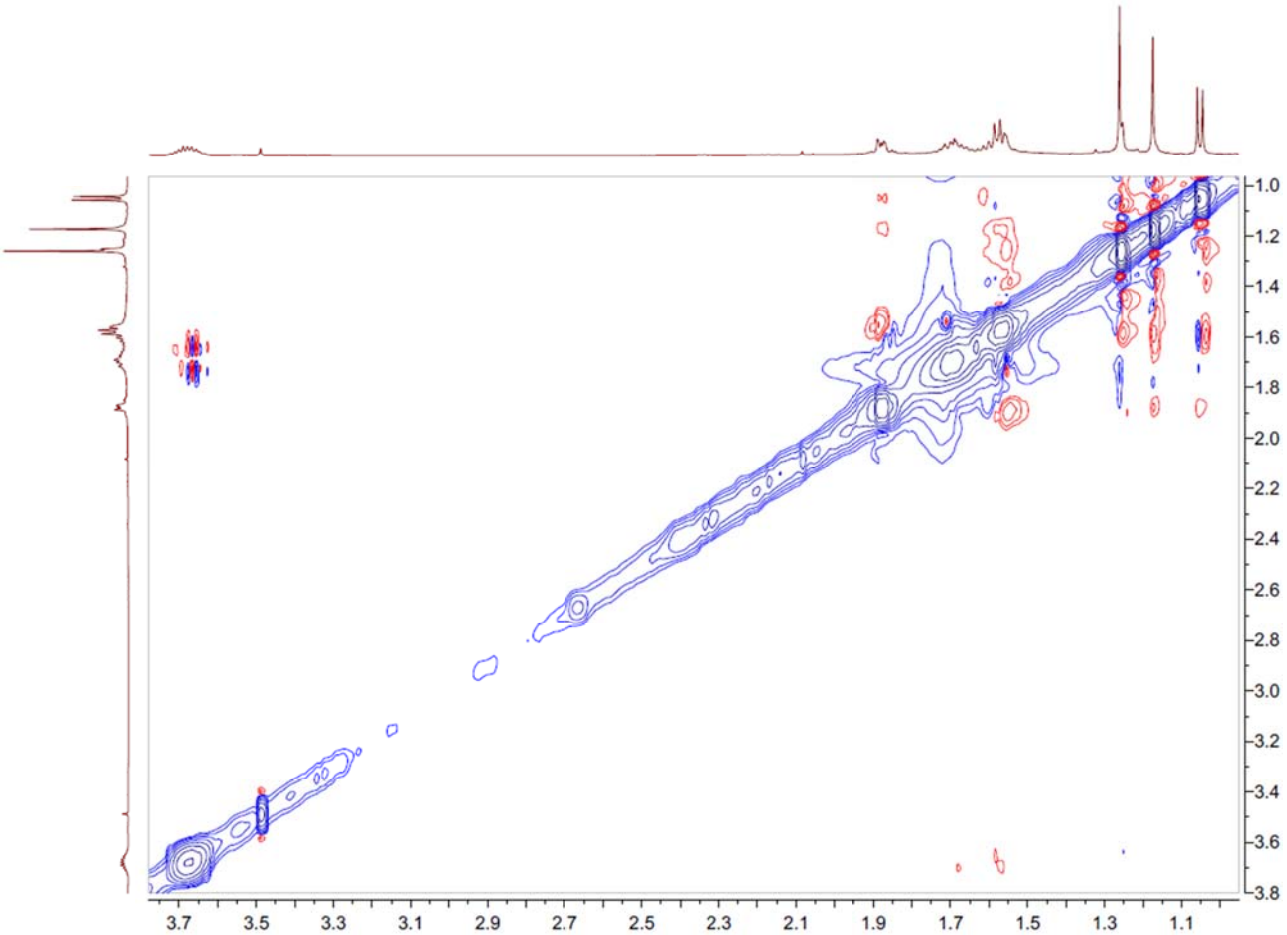

Figure S27. NOESY spectrum of compound **4** in $CDCl_3$.

Single Mass Analysis
Tolerance = 10.0 PPM / DBE: min = -10.0, max = 120.0
Selected filters: None

Monoisotopic Mass, Odd and Even Electron Ions
21 formula(e) evaluated with 1 results within limits (up to 51 closest results for each mass)
Elements Used:
C: 0-200 H: 0-400 O: 2-6

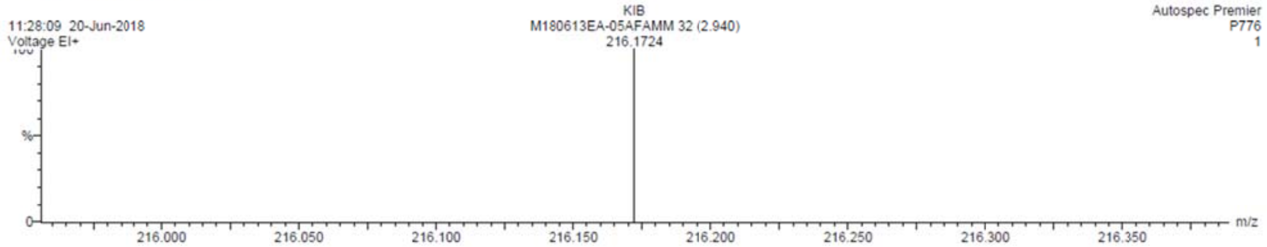

Minimum:				-10.0		
Maximum:		200.0	10.0	120.0		
Mass	Calc. Mass	mDa	PPM	DBE	i-FIT	Formula
216.1724	216.1725	-0.1	-0.5	1.0	5546026.0	C12 H24 O3

Figure S28. HREIMS spectrum of compound **4**.

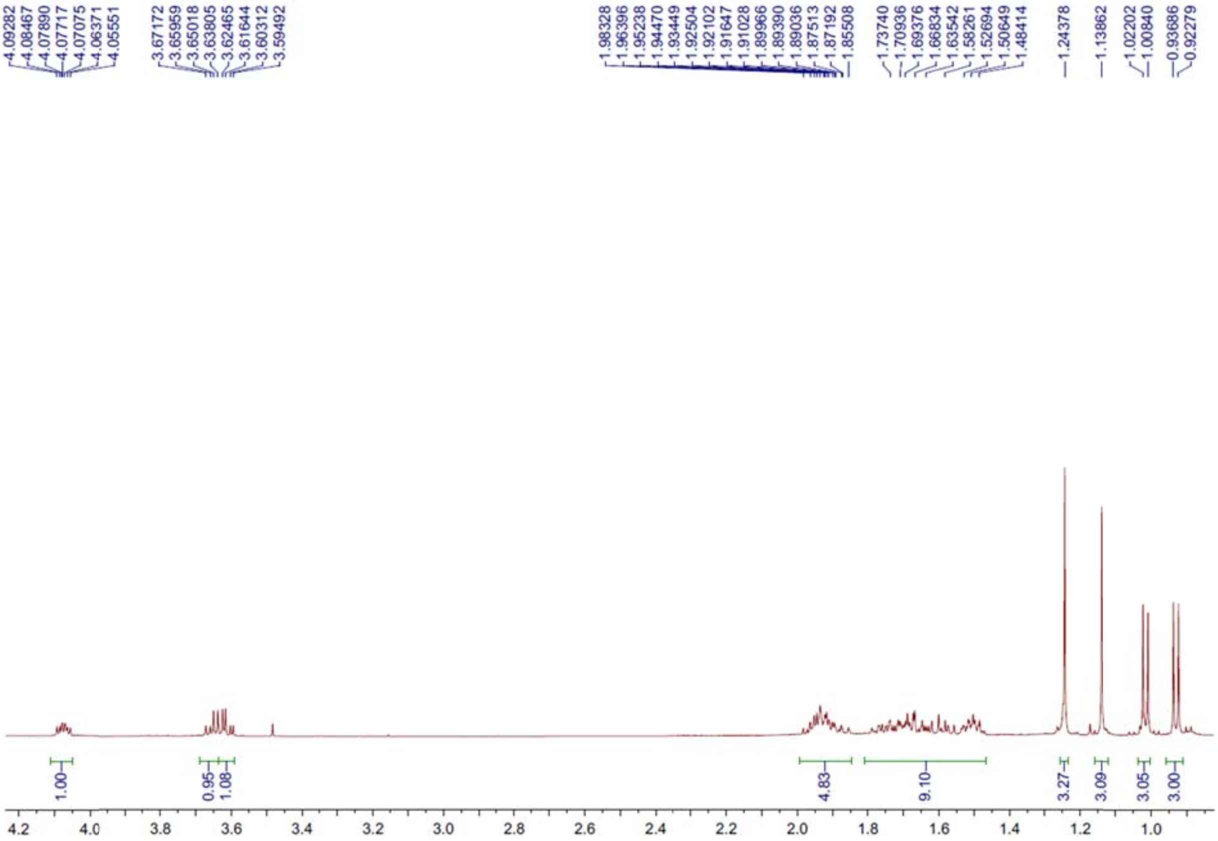

Figure S29. ^{1}H NMR spectrum of compound **5** in $CDCl_3$.

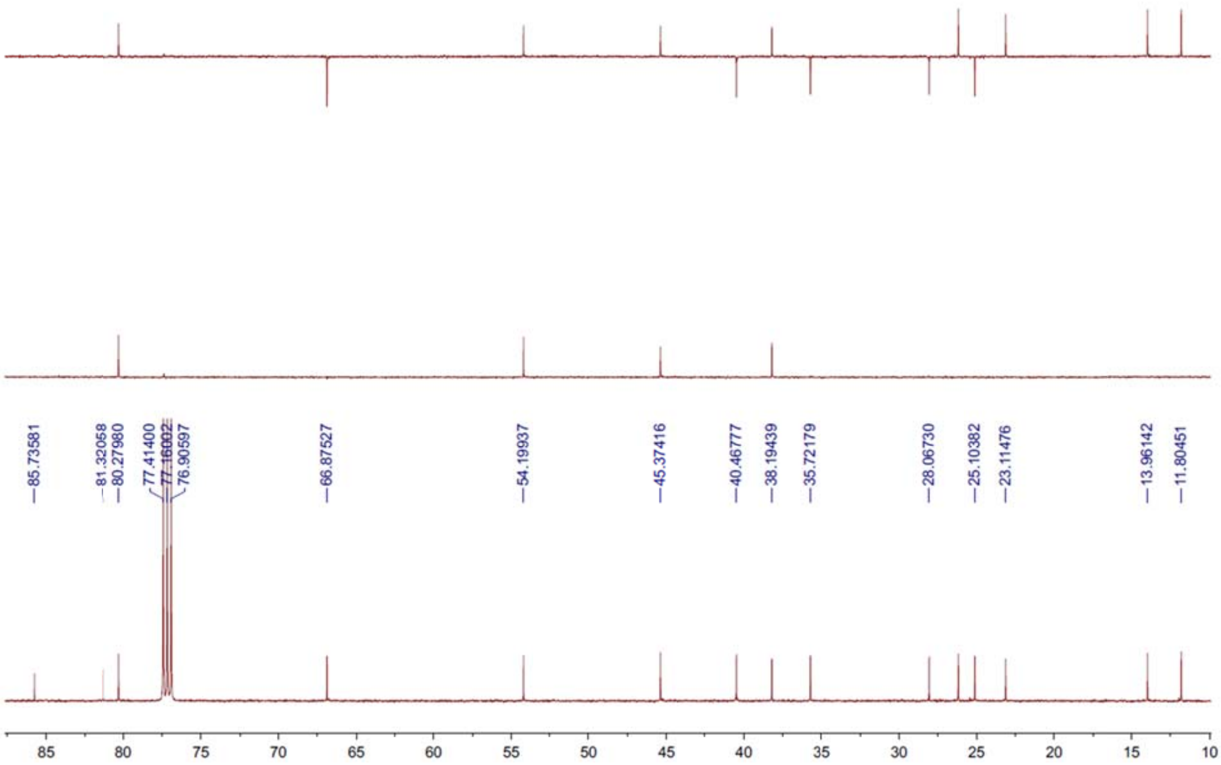

Figure S30. ^{13}C NMR and DEPT spectra of compound **5** in $CDCl_3$.

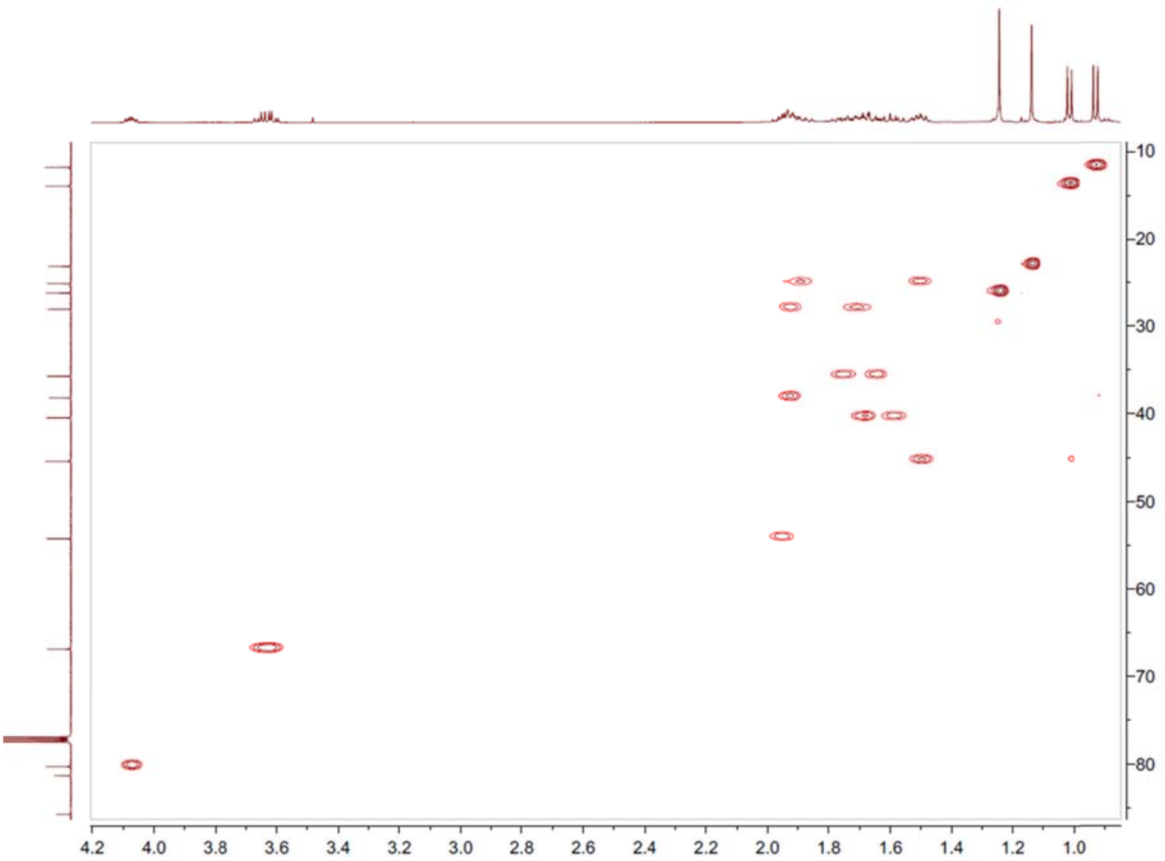

Figure S31. HSQC spectrum of compound **5** in $CDCl_3$.

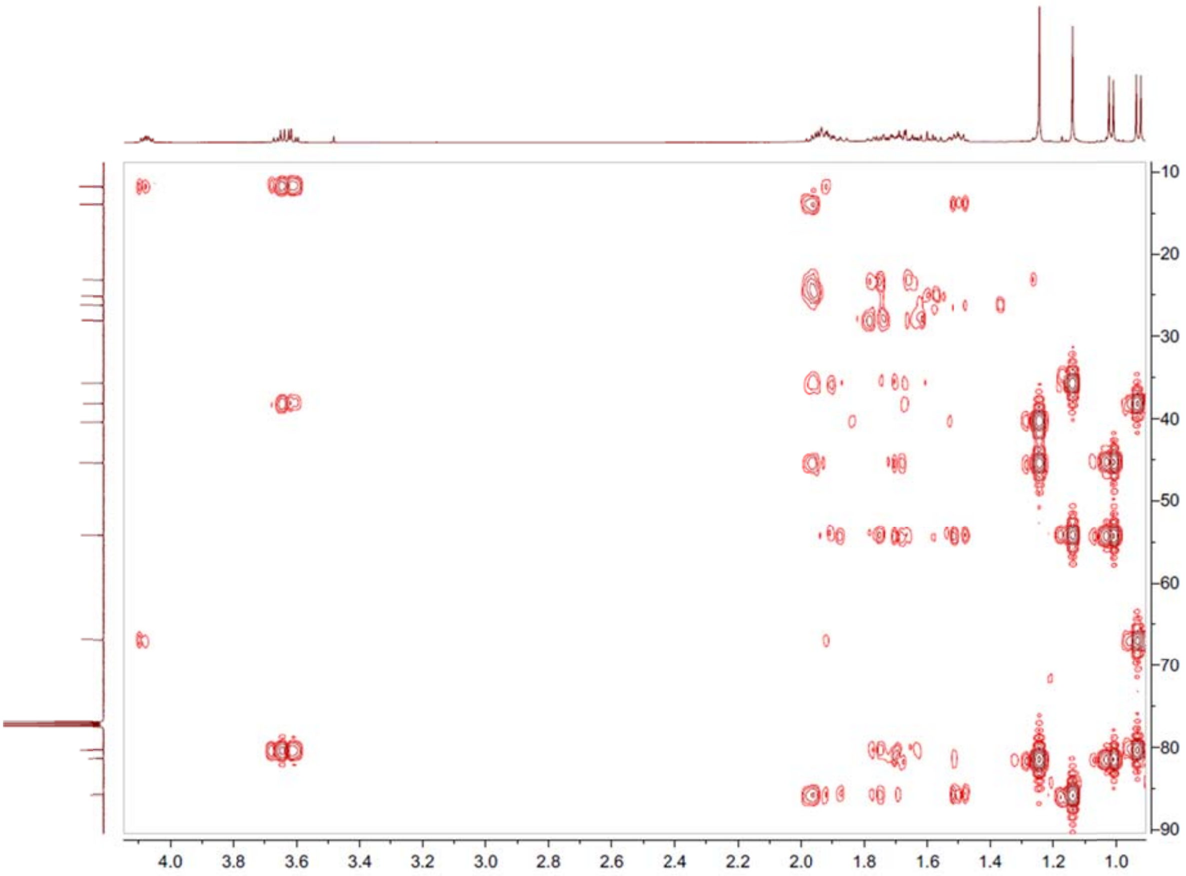

Figure S32. HMBC spectrum of compound **5** in $CDCl_3$.

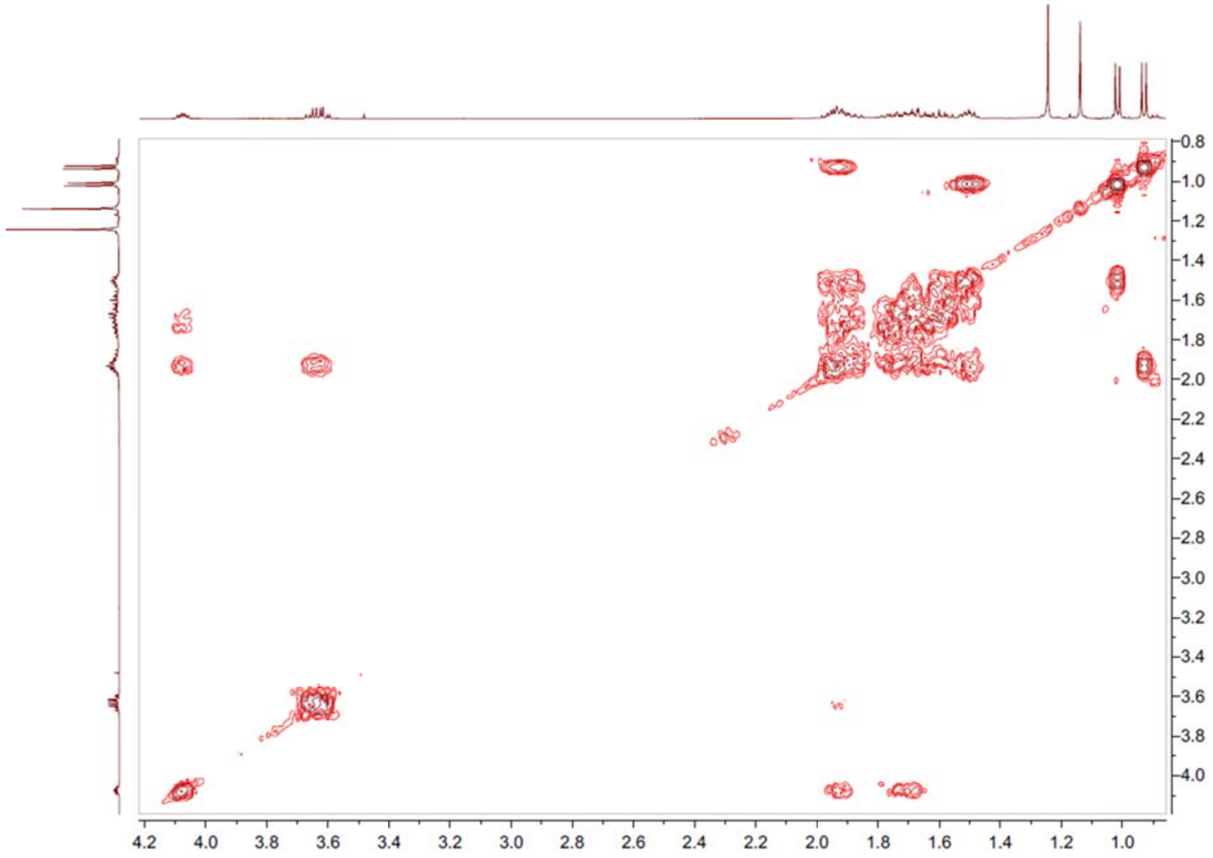

Figure S33. COSY spectrum of compound **5** in $CDCl_3$.

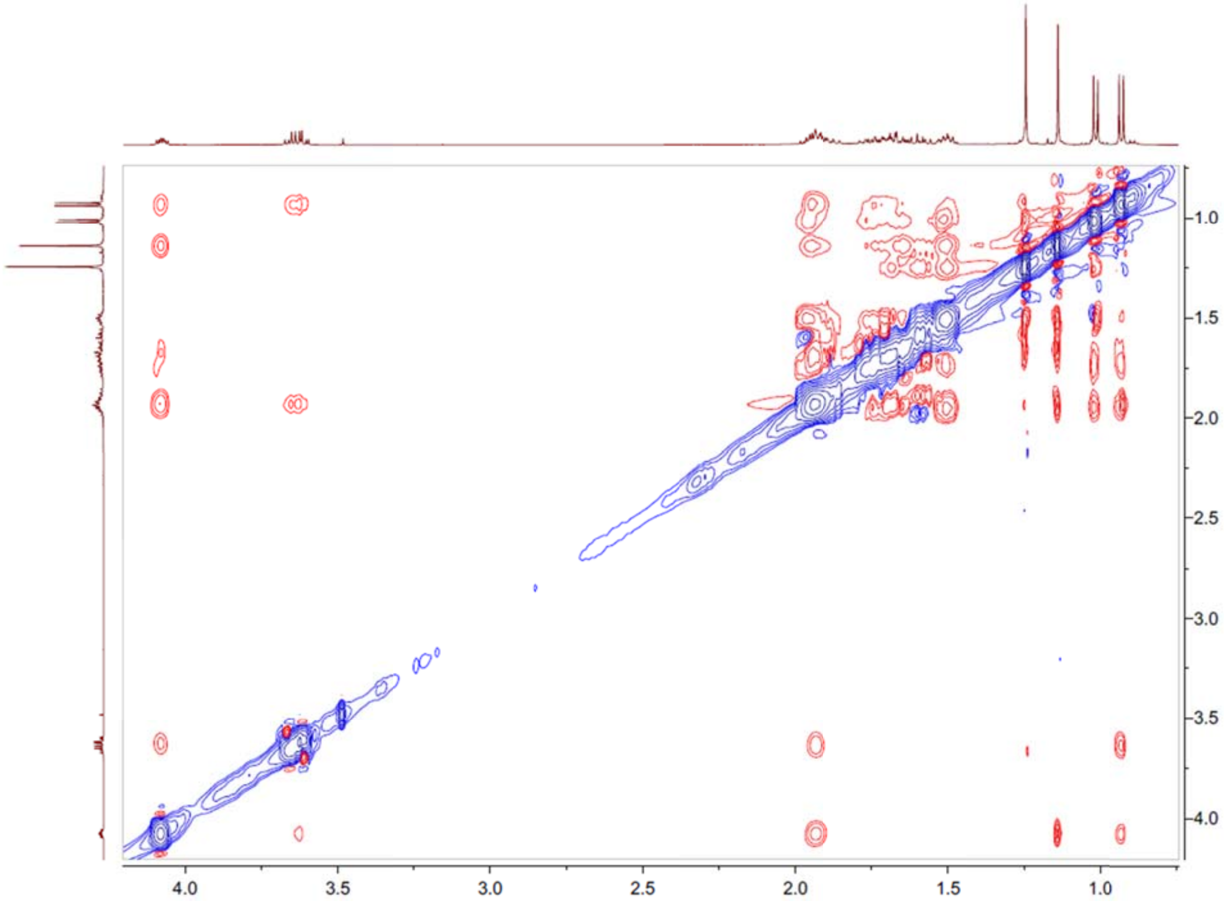

Figure S34. NOESY spectrum of compound **5** in $CDCl_3$.

Single Mass Analysis
Tolerance = 10.0 PPM / DBE: min = -10.0, max = 120.0
Selected filters: None

Monoisotopic Mass, Odd and Even Electron Ions
19 formula(e) evaluated with 1 results within limits (up to 51 closest results for each mass)
Elements Used:
C: 0-200 H: 0-400 O: 2-5

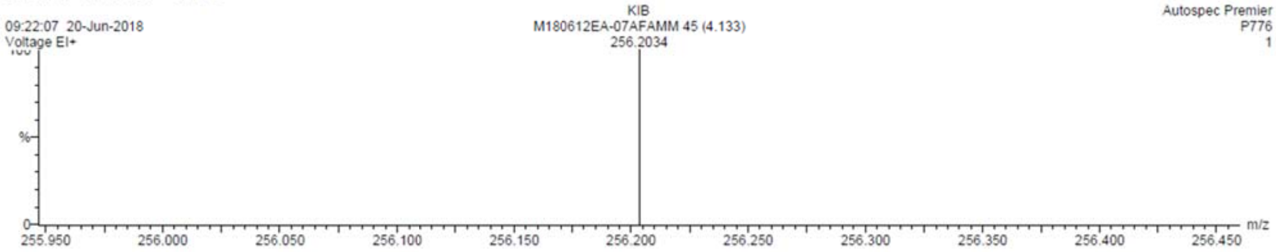

Minimum:				-10.0		
Maximum:		200.0	10.0	120.0		
Mass	Calc. Mass	mDa	PPM	DBE	i-FIT	Formula
256.2034	256.2038	-0.4	-1.6	2.0	5546026.0	C15 H28 O3

Figure S35. HREIMS spectrum of compound **5**.

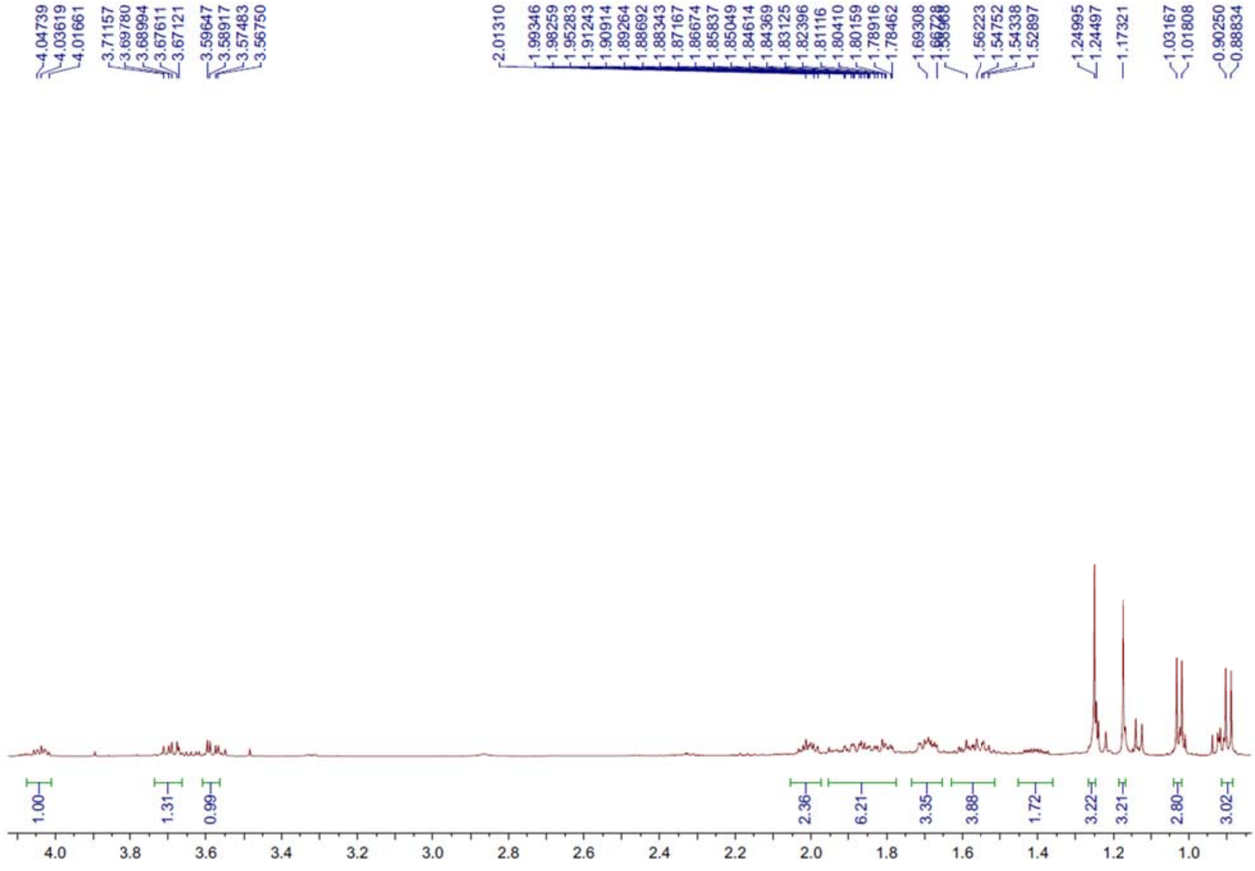

Figure S36. ^{1}H NMR spectrum of compound **6** in $CDCl_3$.

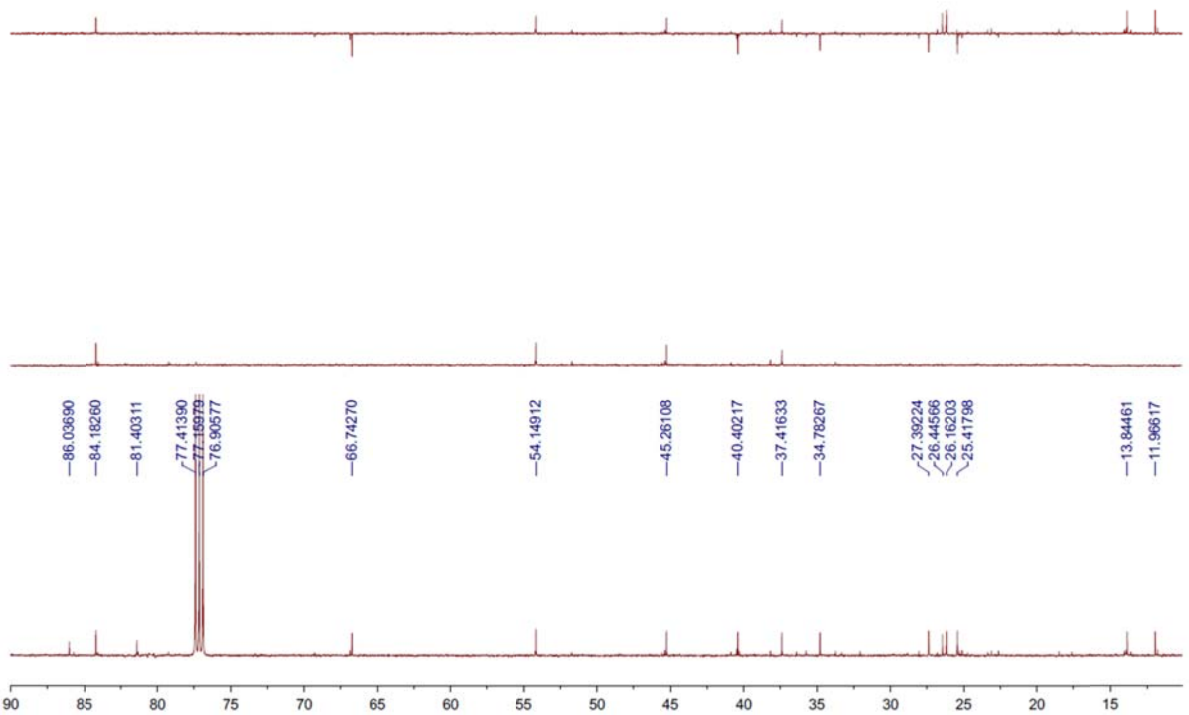

Figure S37. ^{13}C NMR and DEPT spectra of compound **6** in $CDCl_3$.

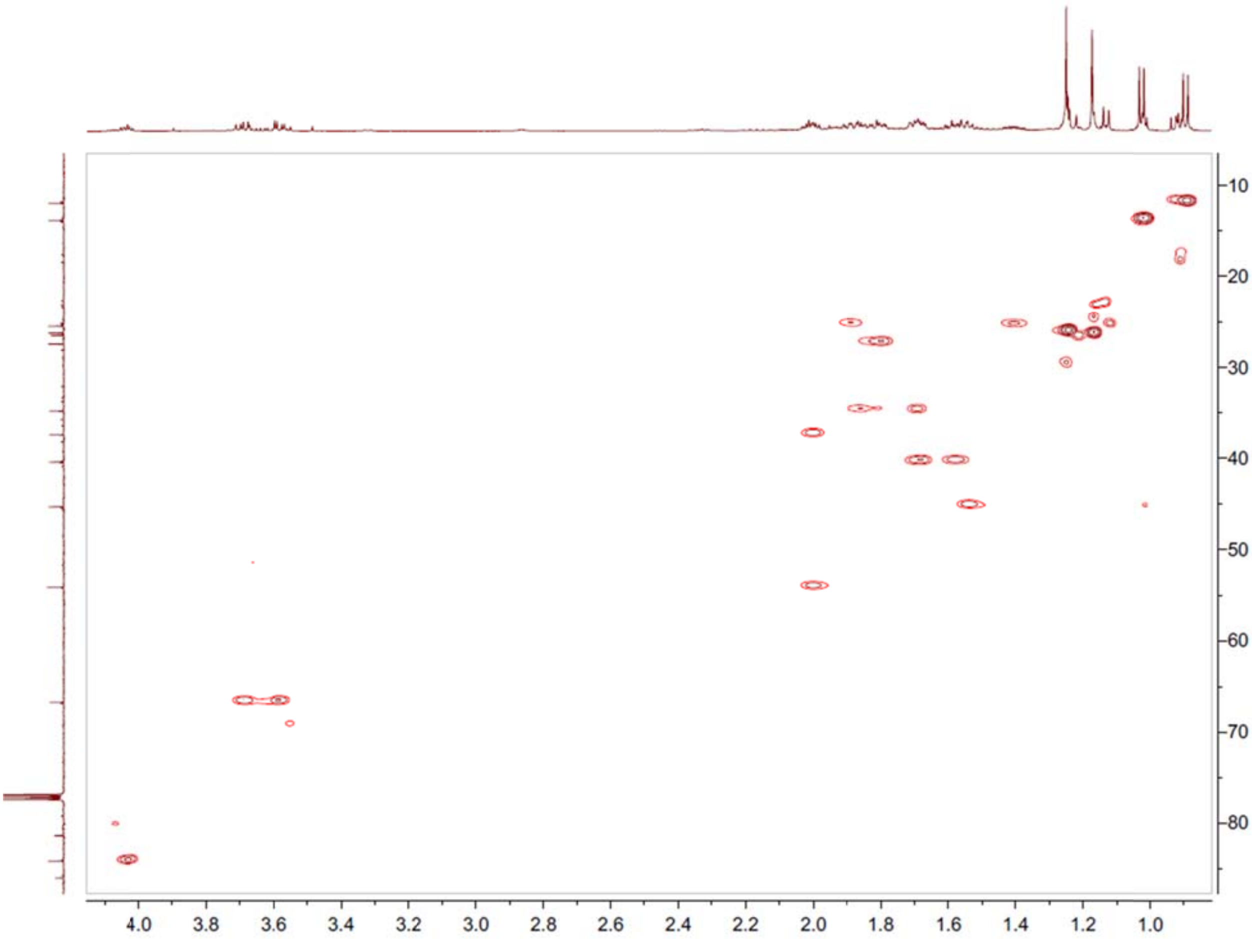

Figure S38. HSQC spectrum of compound **6** in $CDCl_3$.

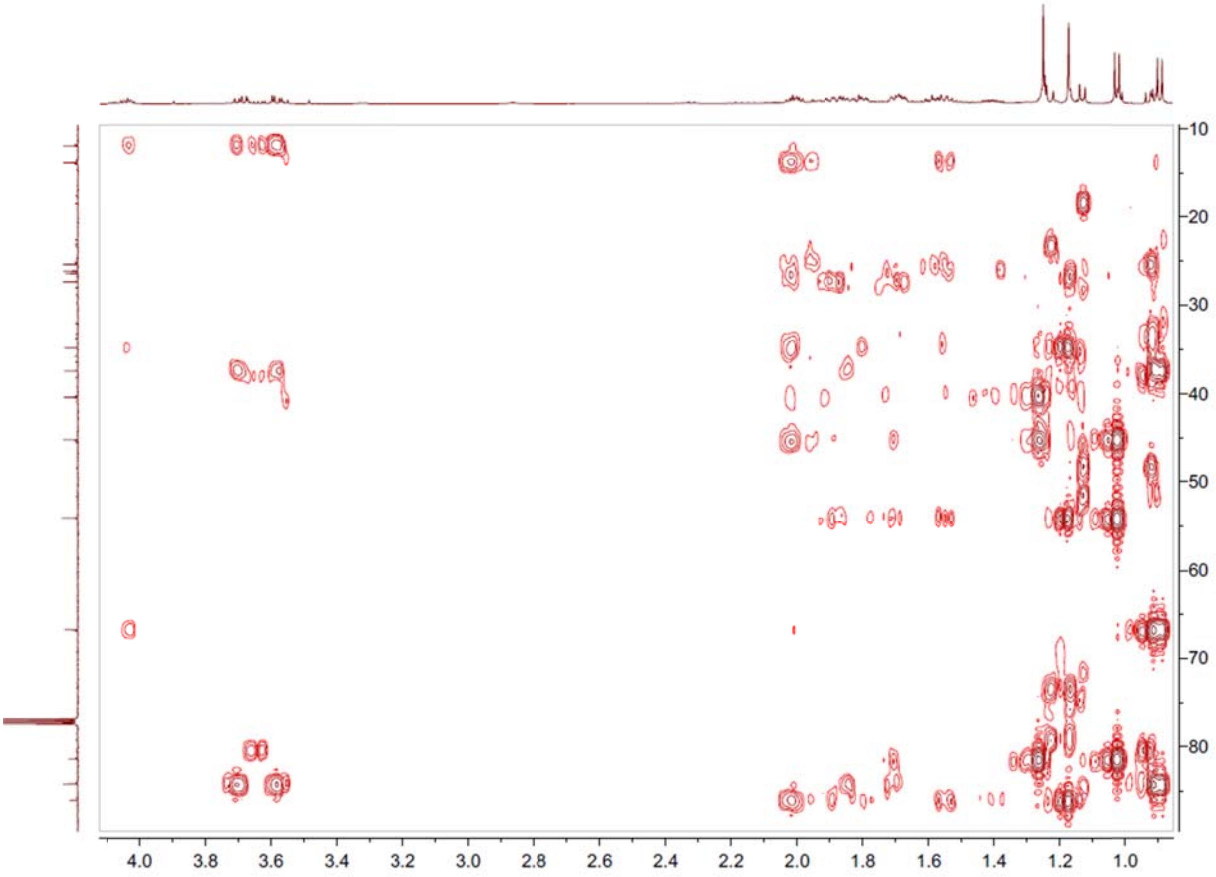

Figure S39. HMBC spectrum of compound **6** in $CDCl_3$.

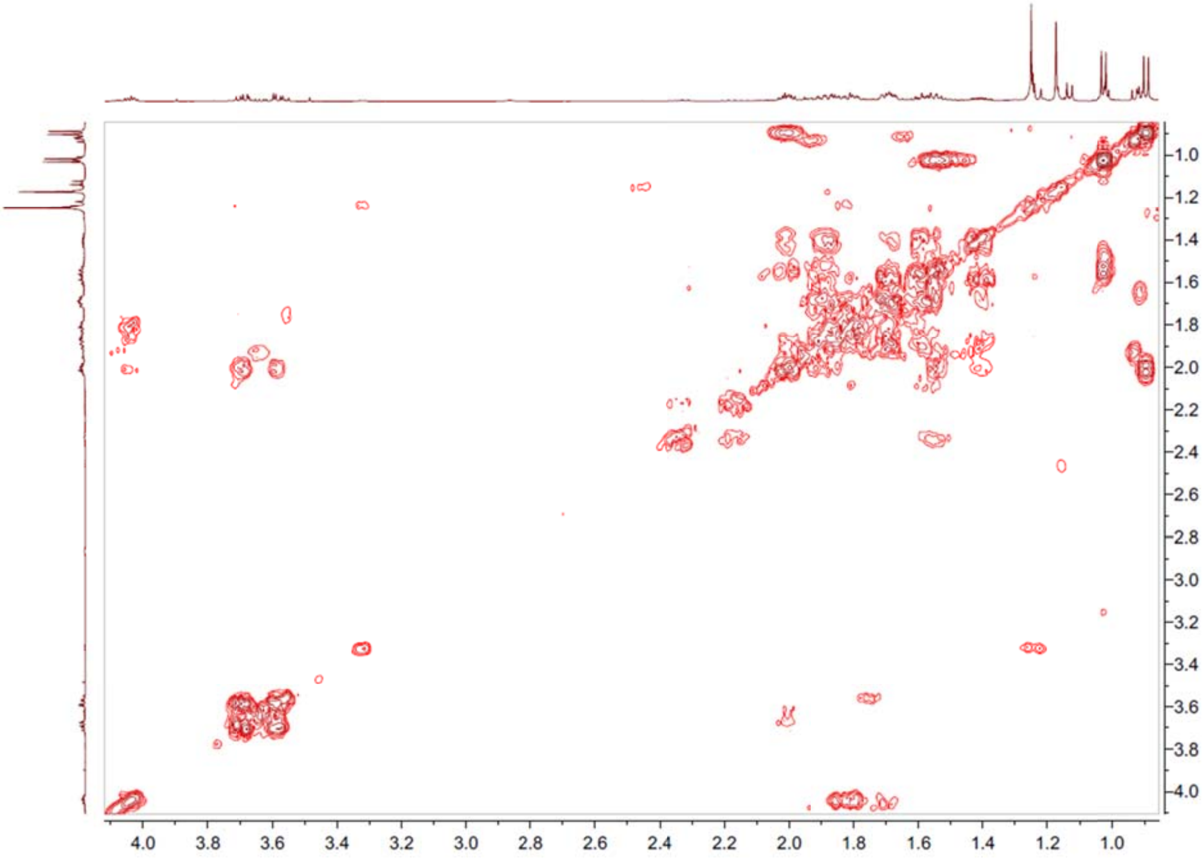

Figure S40. COSY spectrum of compound **6** in $CDCl_3$.

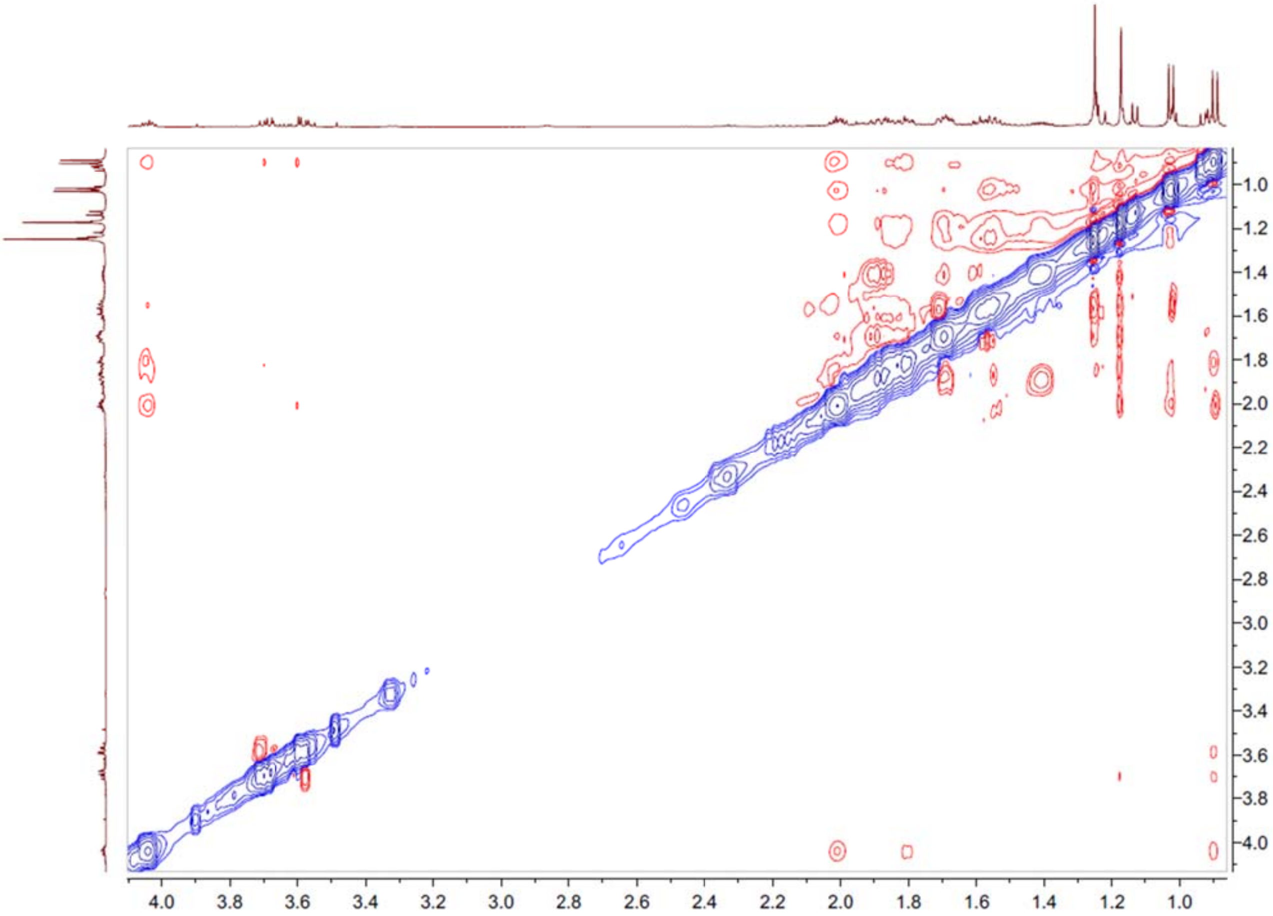

Figure S41. NOESY spectrum of compound **6** in $CDCl_3$.

Single Mass Analysis
Tolerance = 10.0 PPM / DBE: min = -10.0, max = 120.0
Selected filters: None

Monoisotopic Mass, Odd and Even Electron Ions
19 formula(e) evaluated with 1 results within limits (up to 51 closest results for each mass)
Elements Used:
C: 0-200 H: 0-400 O: 2-5

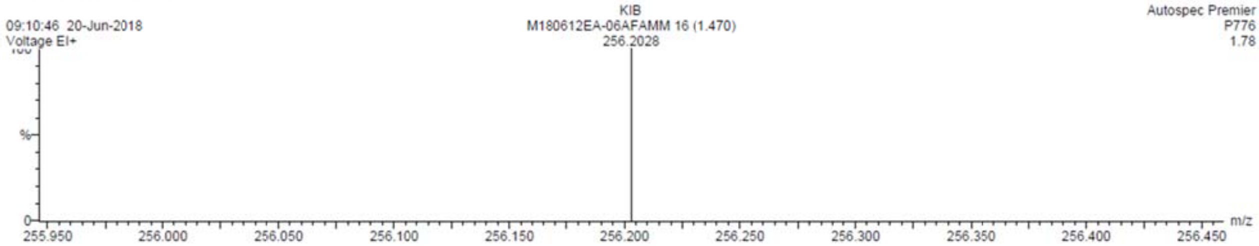

Minimum:				-10.0		
Maximum:		200.0	10.0	120.0		
Mass	Calc. Mass	mDa	PPM	DBE	i-FIT	Formula
256.2028	256.2038	-1.0	-3.9	2.0	5546025.5	C15 H28 O3

Figure S42. HREIMS spectrum of compound **6**.

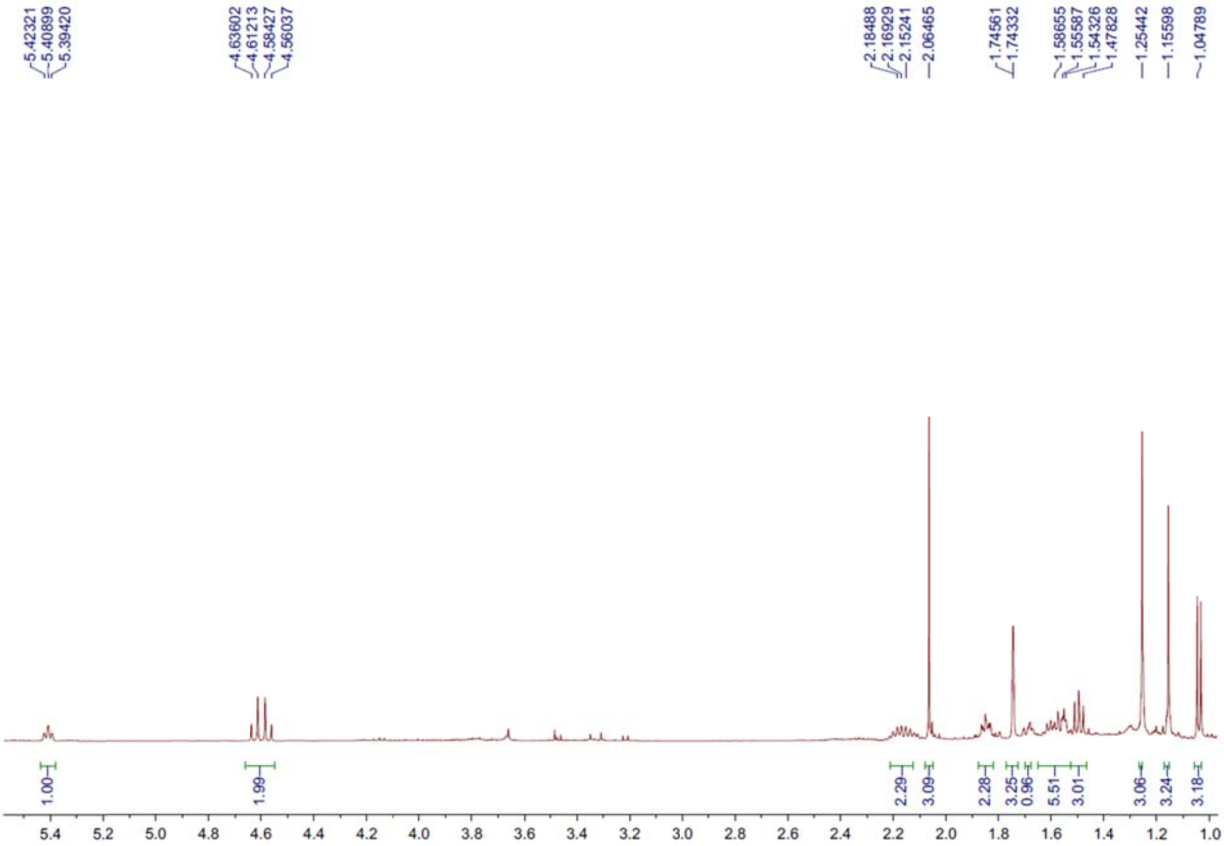

Figure S43. ^{1}H NMR spectrum of compound **7** in $CDCl_3$.

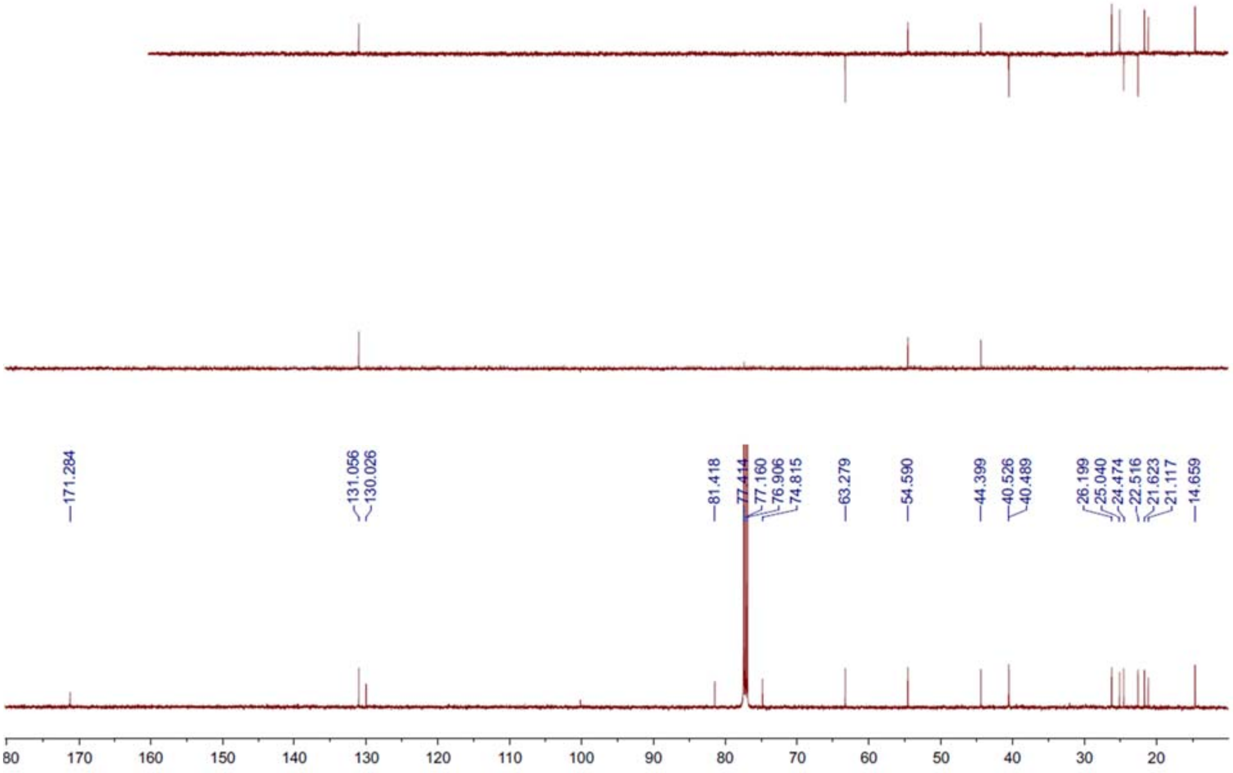

Figure S44. ^{13}C NMR and DEPT spectra of compound **7** in $CDCl_3$.

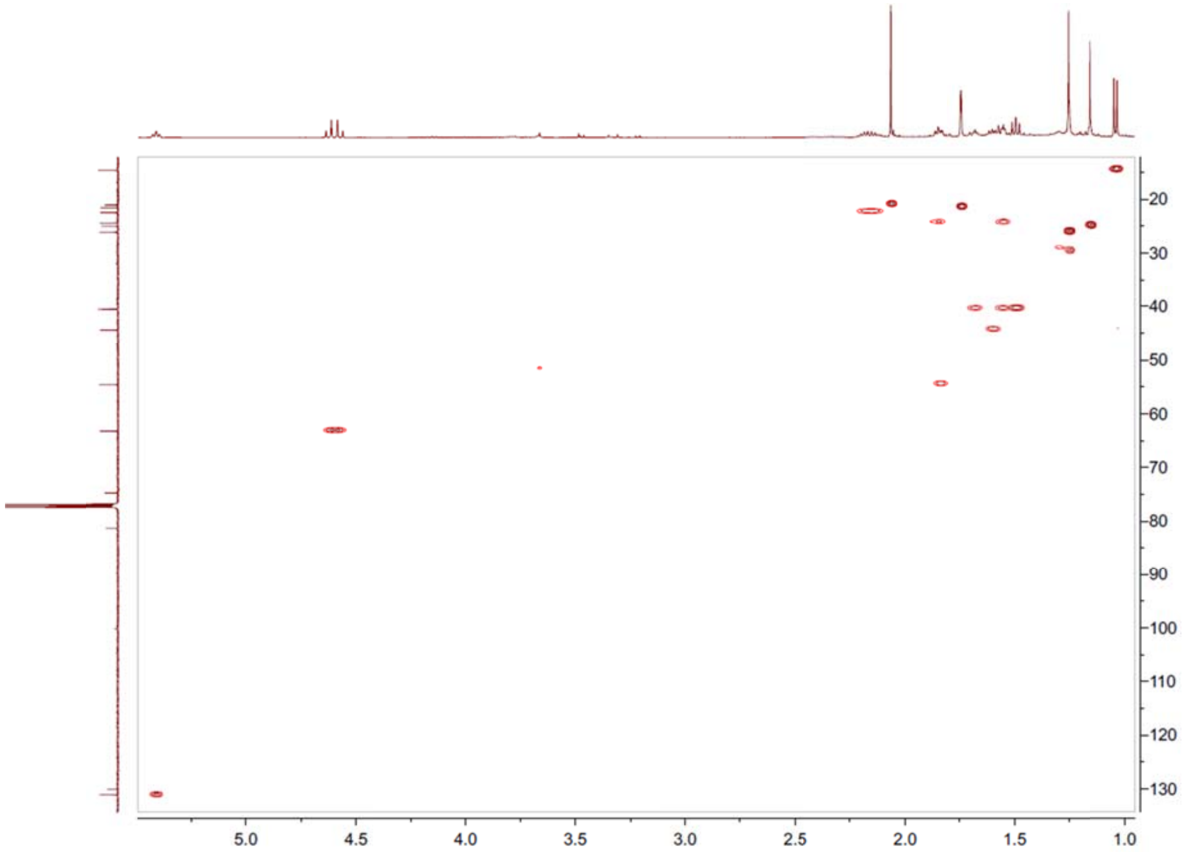

Figure S45. HSQC spectrum of compound **7** in $CDCl_3$.

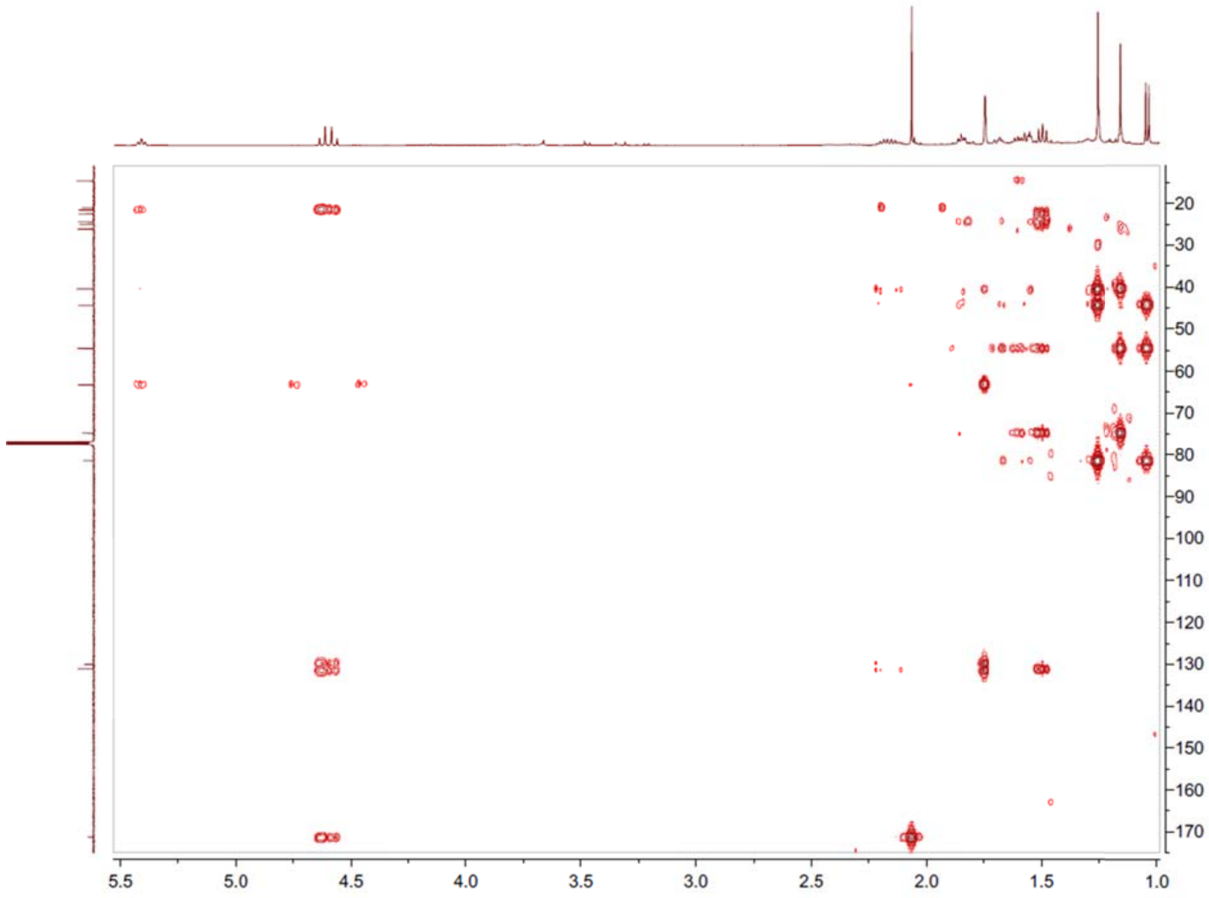

Figure S46. HMBC spectrum of compound **7** in $CDCl_3$.

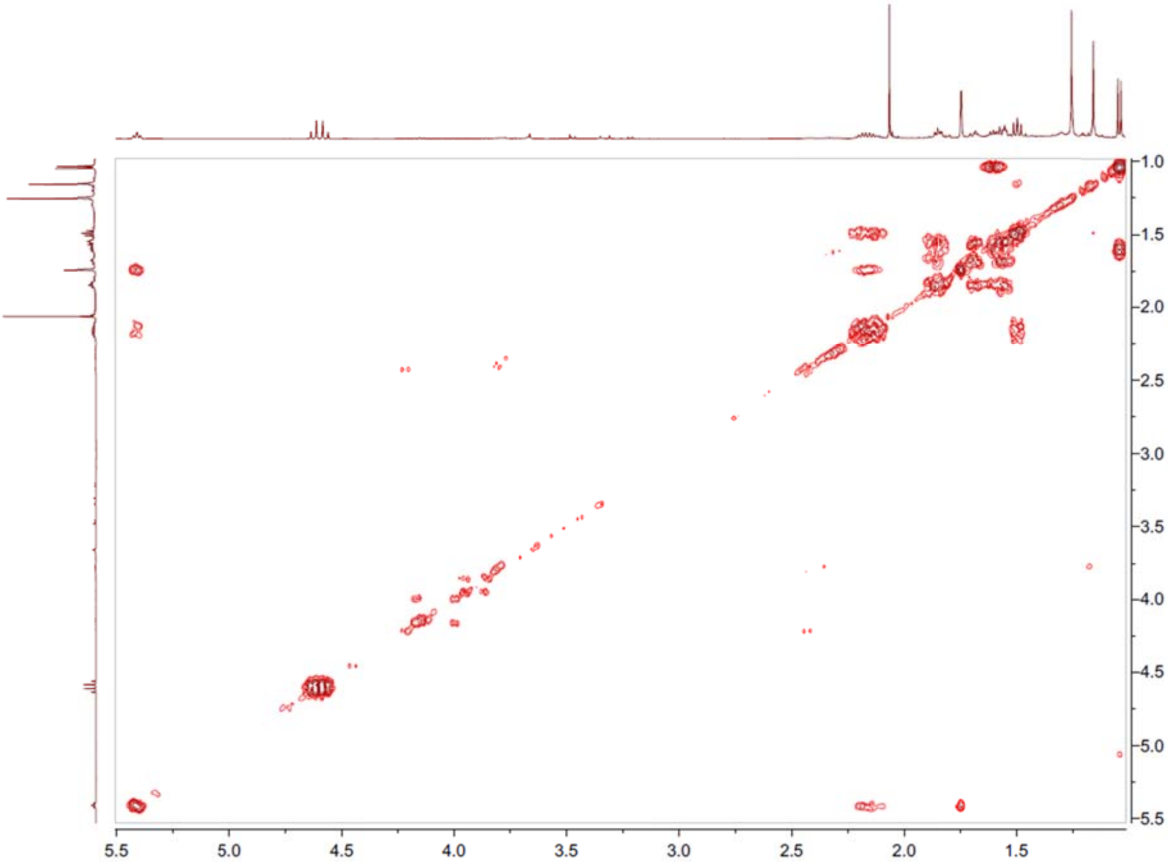

Figure S47. COSY spectrum of compound **7** in $CDCl_3$.

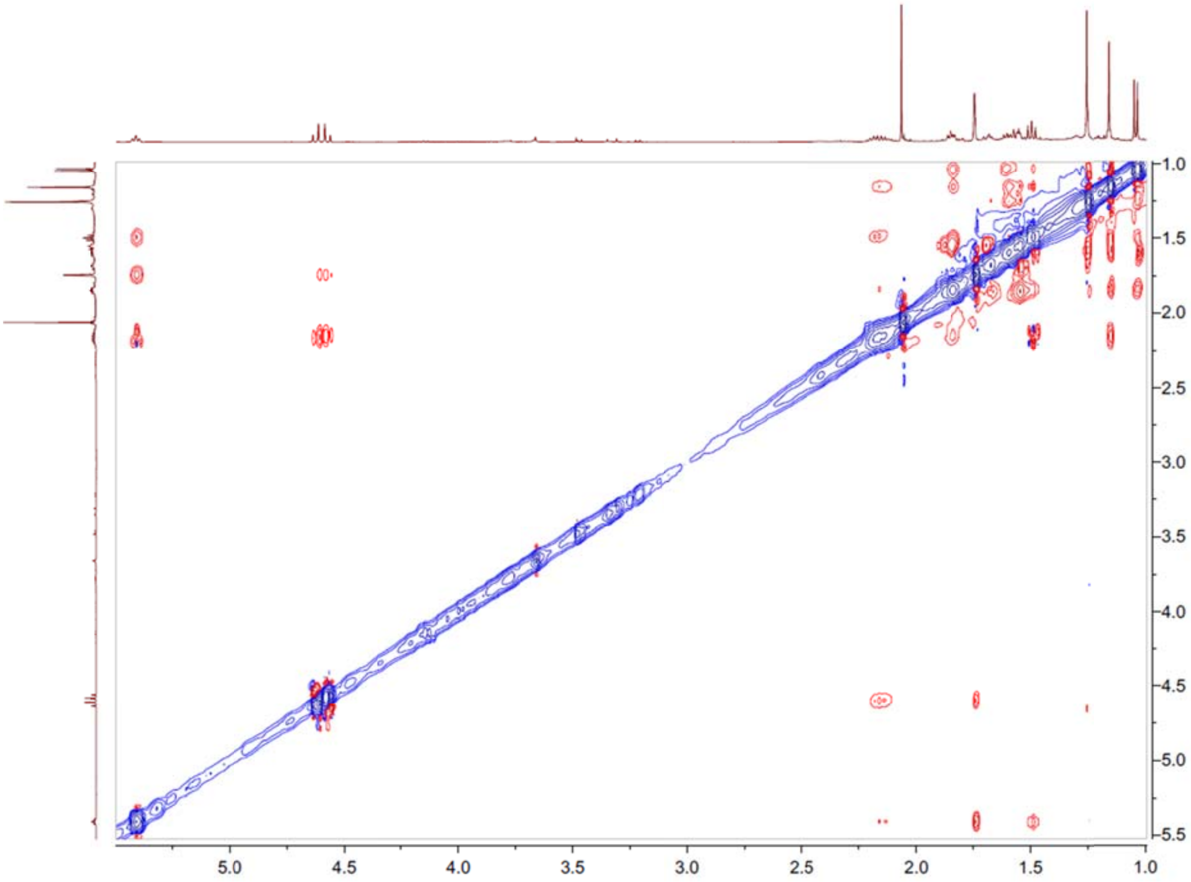

Figure S48. NOESY spectrum of compound **7** in $CDCl_3$.

Single Mass Analysis
Tolerance = 10.0 PPM / DBE: min = -10.0, max = 120.0
Selected filters: None

Monoisotopic Mass, Odd and Even Electron Ions
25 formula(e) evaluated with 1 results within limits (up to 51 closest results for each mass)
Elements Used:
C: 0-200 H: 0-400 O: 2-6

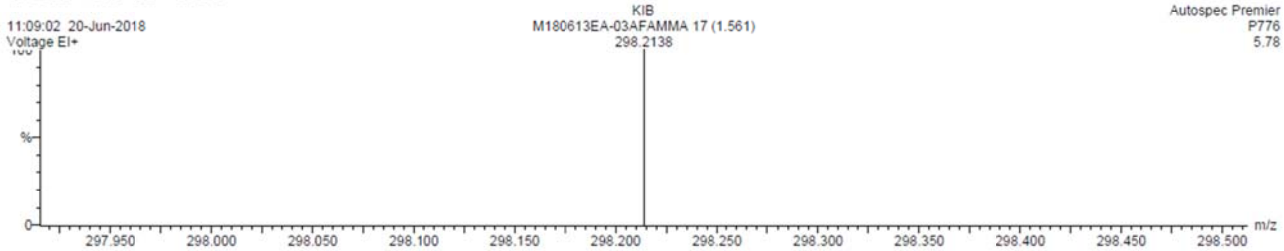

Minimum:				-10.0		
Maximum:		200.0	10.0	120.0		
Mass	Calc. Mass	mDa	PPM	DBE	i-FIT	Formula
298.2138	298.2144	-0.6	-2.0	3.0	5546025.5	C17 H30 O4

Figure S49. HREIMS spectrum of compound **7**.
